# Joint Family and Work Trajectories and Multidimensional Wellbeing

**DOI:** 10.1007/s10680-021-09583-3

**Published:** 2021-04-14

**Authors:** C. L. Comolli, L. Bernardi, M. Voorpostel

**Affiliations:** 1 University of Lausanne, Lausanne, Switzerland; 2 FORS (Swiss Centre of Expertise in the Social Sciences), Lausanne, Switzerland

**Keywords:** Subjective wellbeing, Relational wellbeing, Financial wellbeing, Family trajectories, Professional trajectories, Sequence analysis

## Abstract

Informed by the life course perspective, this paper investigates whether and how employment and family trajectories are jointly associated with subjective, relational and financial wellbeing later in life. We draw on data from the Swiss Household Panel which combines biographical retrospective information on work, partnership and childbearing trajectories with 19 annual waves containing a number of wellbeing indicators as well as detailed socio-demographic and social origin information. We use sequence analysis to identify the main family and work trajectories for men and women aged 20–50 years old. We use OLS regression models to assess the association between those trajectories and their interdependency with wellbeing. Results reveal a joint association between work and family trajectories and wellbeing at older age, even net of social origin and pre-trajectory resources. For women, but not for men, the association is also not fully explained by proximate (current family and work status) determinants of wellbeing. Women’s stable full-time employment combined with traditional family trajectories yields a subjective wellbeing premium, whereas childlessness and absence of a stable partnership over the life course is associated with lower levels of financial and subjective wellbeing after 50 especially in combination with a trajectory of weak labour market involvement. Relational wellbeing is not associated with employment trajectories, and only weakly linked to family trajectories among men.

## Introduction

In the last decades in contemporary societies, both employment and family trajectories have become more diverse and uncertain (Diewald et al., 2006). Many studies show that both the rise in non-standard employment histories and the increasing complexity and multiplicity of family arrangements generate important implications for individuals’ wellbeing and contribute to growing inequality (Barbieri, 2009; Kovalenko & Mortelmans, 2014). Overall, family trajectories characterized by early family formation and unstable partnership histories (Demey et al., 2014; Peters & Liefbroer, 1997; Zimmermann & Hameister, 2019) and work trajectories characterized by non-employment (Falkingham et al., 2020; Ponomarenko, 2016) tend to be associated with lower wellbeing later in life, compared with delayed family formation and a strong attachment to the labour market.

As the rich literature on the spillover between the work and family domains demonstrates (Charles & Stephens, 2004), the two have also become more intertwined, given the simultaneous increase in the share of dual earner couples and in the demands of both the parent’s and worker’s roles (Drobnič & Guillén, 2011; Van der Lippe & Peters, 2007). However, despite the advantages of studying the combined patterns of employment and family arrays have been highlighted earlier (Aassve, Billari, et al., 2007; McDonough et al., 2015), and their joint impact on wellbeing, and their interplay, is still rarely addressed in the literature, especially adopting a holistic and multidimensional perspective (Abbot, 2005). In a holistic view, the life course is a process in which events and transitions occur in a continuum, shifting the focus from single events or transitions to long-term work and family trajectories (Elder, 2001; Piccarreta & Studer, 2019: pp. 1). The multidimensionality of the life course refers both to the simultaneous look at multiple domains and to the assessment of the influence of such life course trajectories on various wellbeing dimensions (Bernardi et al., 2019).

We build on recent studies showing that some types of work and family trajectories generate greater vulnerability in later life (McDonough et al., 2015) in terms of health (Arpino et al., 2018; Lacey et al., 2017; Lacey, Sacker et al., 2016), lower life satisfaction (Lacey, Stafford et al., 2016; Schmalzle et al., 2019) and financial wellbeing (Halpern-Manners et al., 2015; Madero-Cabib & Fasang, 2016). Our study is unique in measuring the extent to which early to mid-adulthood employment–family trajectories are *jointly* related to subjective, relational and financial wellbeing later on (Bernardi et al., 2019) net of pre-trajectory conditions and more proximal determinants of wellbeing. In particular, our investigation is guided by three research questions. First, we ask whether work and family trajectories interplay in influencing multiple dimensions of wellbeing later on. Second, we ask whether the association between joint work–family trajectories and wellbeing later in life is explained by early disadvantages and proximate determinants of wellbeing. Early socio-economic conditions (such as family of origin characteristics) have been shown to shape both the likelihood of individuals experiencing a certain work–family trajectory and to affect how critical transitions are related to wellbeing (Arpino et al., 2018; Schafer et al., 2013). Moreover, family and work trajectories and later wellbeing are directly associated, beyond the joint work–family trajectories, through the family and employment status respondents hold when wellbeing is measured. Finally, we compare the association between trajectories and wellbeing between men and women, given that the process linking family and work histories to wellbeing is likely to be gendered: trajectories differ by gender, and the work and life domains generally are less reconcilable for women (Keizer et al., 2010).

We draw on data from the large-scale, nationally representative longitudinal Swiss Household Panel (SHP), using a subsample that completed a biographical retrospective calendar covering complete work and family trajectories prior to entering the panel. This allows us to identify the critical family transitions of parenthood, partnering and re-partnering after a union dissolution, and on the critical employment transitions from school to work and in and out of joblessness. The SHP also contains a variety of indicators of wellbeing recorded yearly. We use sequence analysis to identify and describe the main trajectories of work and family of the respondents based on the biographical data and estimate linear regression models to assess the association between these combined trajectories and the wellbeing outcomes.

## Background

### Family and Work Trajectories and Wellbeing

Family and employment trajectories are both independently related to wellbeing outcomes. Long-term stable partnerships bring emotional support and social integration as well as financial and material benefits (Gerstel et al., 1985). Stable unions tend to be associated with greater life satisfaction (Thomson et al., 2001) and less loneliness in later life (Peters & Liefbroer, 1997). In contrast, trajectories characterized by (multiple) union dissolutions and absence of a partner tend to be linked to lower affective, subjective and social wellbeing (Demey et al., 2014; Zimmermann & Hameister, 2019) but also lower economic wellbeing (Aassve, Betti et al., 2007; Halper-Manners et al., 2015). On the one hand, childlessness means less access to social resources and support (Nordenmark, 2004) and might still represent a non-normative family type especially for women (Lacey, Stafford et al., 2016). In the long term, childlessness is linked to lower life satisfaction (Hansen et al., 2009). Parenthood tends to induce positive emotions, a sense of meaning and psychological growth and to increase social integration (Roeters et al., 2016). On the other hand, early family formation tends to be linked to lower educational attainment, lower likelihood of full-time employment and lower subjective wellbeing compared with a delayed family formation (Schoon et al., 2012).

Strong labour market attachment provides social networks that are beneficial for relational wellbeing, financial resources as well as opportunities for personal reward and learning (Clark et al., 2001). Career interruptions not only directly reduce life satisfaction (Oesch & Lipps, 2013) but also indirectly affect wellbeing later on, by reducing the accumulation of financial assets and tenure and thus lowering future job prospects (Gangl, 2006), health (Young, 2012) and partnering chances (Amato & Beattie, 2011). While part-time employment might produce scarring effects and a lower probability of re-entering the labour market with full-time employment (Fouarge & Muffels, 2008), evidence on the wellbeing consequences of part-time and late return to part-time work trajectories is mixed and tends to depend on the willingness to work part-time and on the length of the spell (Falkingham et al., 2020). The longer the part-time spell, the more negative the consequences for subjective wellbeing, unless the part-time option is chosen voluntary to reconcile family and work obligations (Ponomarenko, 2016). Finally, compared with full-time employment, also early retirement, self-employment, family caring and atypical work have been linked to lower subjective wellbeing (Falkingham et al., 2020). Subjective wellbeing trajectories after retirement are more positive when the long-term employment pathway to retirement is characterized by full-time work, compared to transitioning into retirement from inactivity or after a trajectory of unemployment (Schmalzle et al., 2019).

### Joint Family and Work Trajectories and Wellbeing

The life course framework stresses the multidimensionality of biographies (Elder, 2001) treating the life course as a set of events and transitions occurring in multiple domains simultaneously (Diewald & Mayer, 2009). In fact, not only work and family trajectories have become more uncertain, the two are also more intertwined than in the past (Aassve, Billari et al., 2007; Drobnič & Guillén, 2011). The increase in female labour force participation has led to an increase in the number of dual earner couples in which the negotiation between partners to balance family and work has become a pressing issue. At the same time, balancing between multiple roles has become harder given the increasing demands from the work place (Van der Lippe & Peters, 2007) and the rising standards of parenting (Jacobs & Gerson, 2004). The result is increased conflict between these two life domains (Matthews et al., 2014) as the rich literature on spillover between the work and family domains shows (Charles & Stephens, 2004). Therefore, in order to fully understand the implications of the increasing complexity of lives in contemporary society, it is paramount to investigate the professional and family spheres together.

Most studies show that individuals with life course trajectories characterized by a strong attachment to the labour market in combination with stable partnership and parenthood tend to display the greatest wellbeing. Lacey et al. (2016b) report that British women who combine marriage and parenthood with little or no long-term ties to the labour market displayed lower subjective wellbeing during retirement age, even when accounting for prior wellbeing. Besides the lack of access to the benefits provided by labour market work, also children leaving the parental home has been previously shown to be more stressful for mothers who do not work (Adelmann et al., 1989). In a recent study, Xue and colleagues (2020) show that trajectories characterized by late transition to both family formation and full-time work lead women to higher subjective wellbeing later on. Madero-Cabib and Fasang (2016) show that when women combine early motherhood with a weak attachment to the labour market their observed financial wellbeing at retirement age is lower than when women have more continued employment trajectories. McDonough et al., (2015) find compensatory mechanisms between the two spheres of work and family life: as much as a history of stable marriage might compensate for a weak labour market attachment among mothers, absence of a partner can be compensated by a trajectory of stable full-time employment. Similarly, Xue et al., (2020) find that childlessness combined with a strong work orientation also leads to sustained wellbeing among women.

### Early (dis)advantages and Proximate Determinants of Wellbeing

Critical events, trajectories and wellbeing are not equally distributed across individuals in society. Embedded in the life course paradigm, the Cumulative Advantage/Disadvantage (CAD) theory posits that individuals experience unique trajectories and outcomes that become increasingly different as individuals age. The benefits associated with a person’s structural position early in the life course—such as social origin or childhood experiences—tend to cumulate over time, through path-dependent processes that generate trajectories that lead to certain outcomes later in life, widening the social difference with other groups as they age (Dannefer, 2018).

Social origin affects life course trajectories and wellbeing both directly and indirectly. Individuals with greater resources, for instance, growing up in higher socio-economic status families or in better health, not only display better wellbeing outcomes (Diener et al., 2010) but they are also less likely to experience more stressful trajectories in both family and work domains (McLanahan, 2004). Multiple studies demonstrate that an advantaged childhood and adolescence socio-economic status in the form of family structure, higher parental education, better housing and health conditions set individuals into own education, work and family trajectories that are more beneficial for later wellbeing and health outcomes (Arpino et al., 2018; Falkingham et al., 2020; Schafer et al., 2013).

Not only do those pathways influence later-life outcomes directly, but early experiences also influence later outcomes indirectly through more proximal determinants (Bongaarts, 1978), namely the mid-to-late life opportunities they generate. While most previous studies tended to assume that personal biographies become irrelevant for wellbeing once more proximal indicators of work and family circumstances are taken into consideration (Gustman et al. 1996), Halpern-Manners and colleagues (2015) demonstrate that work and family trajectories have both a direct effect on later-life economic wellbeing and an indirect effect through more proximate measures of work and family circumstances.

### Gender Differences

The process linking family–work trajectories to wellbeing is gendered. While partnership trajectories have become more complex for both men and women, women’s work trajectories have become more similar to men’s trajectories (Keizer et al., 2010; Melchior et al., 2007), making the reconciliation of the two domains more complicated for women (Moen & Sweet, 2004). Women’s increasing participation on the labour market in the last decades has been in many countries largely concentrated on part-time jobs, especially among mothers (Ernst Stähli et al., 2009) and career breaks remain more common among women (Ponomarenko, 2016). While the latter expose women more to financial insecurity than men, through more uncertainty and job instability, lower wages and fewer career opportunities and benefits, evidence on subjective wellbeing is mixed, with some studies showing that unemployment and inactivity have larger negative consequences for life satisfaction among men (Ponomarenko, 2016). Moreover, if part-time work is stable and seen as a voluntary strategy to reconcile motherhood and labour market participation, it might lead to greater wellbeing in the long term (Ponomarenko, 2016).

Family formation tends to take place earlier in the life course for women than men (Bruckner and Mayer 2004) which often leads to poor education and a weaker attachment to the labour market and lower subjective wellbeing (Schoon et al., 2012). In case of divorce or separation, women re-marry less frequently than men (de Graaf & Kalmijn, 2003). Men have been shown to benefit more than women from stable unions in terms of life style and wellbeing, and to suffer more from extended periods as single, in terms of overall and relational wellbeing. Women tend to suffer being unpartnered less than men because they value more their independence and cultivate larger networks of family and friends that compensate the lack of partner (Baumbusch, 2004). Unstable union histories instead have worse consequences for women than men in terms of subjective wellbeing and loneliness (Demey et al., 2014; Peters & Liefbroer, 1997; Zimmermann & Hameister, 2019).

Despite the rapid increase in women’s labour force participation, work practices are still largely designed based on a predominantly male workforce, without childcare or domestic work (Moen & Sweet, 2004). Dual-earner couples’ strategy to reconcile work and family is to give priority to men’s career, making women’s career secondary. While men’s work tends to remain more isolated from family responsibilities, women accommodate working time to family needs when needed (Moen, 2018; Moen & Sweet, 2004), which tend to produce overall more negative consequences for women than men.

### Multidimensional Wellbeing

Wellbeing is a multi-faceted concept, including multiple dimensions that are strongly related (Chavez et al., 2005). Some see the relationship between such dimensions as reflecting a unique underlying overall wellbeing evaluation mostly determined by temperamental predispositions (Diener, 1984; Diener & Lucas, 1999). Others think of each dimension as reflecting the objective circumstances individuals experience in the specific domain they refer to (Blanchflowers and Oswald, 2011). The life domain approach (Campbell et al., 1976) sees subjective wellbeing as the net outcome of satisfaction with various life domains. Life satisfaction, the cognitive aspect of subjective well-being (Diener, 1984), is an aggregate measure of satisfaction in various life domains such as work, finances, relationships or leisure activities (Bernardi et al., 2017; Diener et al., 2003). Domain-specific wellbeing indicators reflect the distance between goals, needs and aspirations—subjective factors—and the objective circumstances in each domain (Stone et al., 2010). In a bottom-up process, individuals evaluate separately each domain and each specific evaluation influences overall life satisfaction (McAdams et al., 2012; Schimmack, 2008). It might be that, in relation to specific life-course events, satisfaction in some life domains change in positive direction, while satisfaction in other domains decreases—in a compensatory way—or that particular events trigger positive or negative changes in different domains at the same time—in a cumulative way (Diewald, 2003). Additionally, personal characteristics such as age, health conditions or past experiences also influence the evaluation of the satisfaction in different life domains so that differences between individuals with similar family, work or financial status can still emerge.

In line with a life domain approach, we understand wellbeing as a multidimensional concept and investigate the extent to which long-term joint employment–family trajectories are related not only to overall subjective wellbeing, but to two domain-specific wellbeing indicators: relational and financial wellbeing. A given family–work history might be associated with a lower (or higher) life satisfaction because that trajectory lowers (increases) the satisfaction with personal relationships and/or because it lowers (increases) financial satisfaction. Investigating these multiple dimensions together allows us to identify whether specific work–family trajectories bear long-term consequences in some but not other domains and whether wellbeing in any particular domain respond similarly to overall subjective wellbeing.

Previous studies identify relational satisfaction as an independent but related component of subjective wellbeing (De Leersnyder et al., 2014; Götz et al., 2018). Baumeister and Leary (1995) maintain that quality of life is enhanced by lasting, positive interpersonal relationships and that the lack of satisfaction with personal relationships puts individuals at risk of loneliness and lower subjective wellbeing (Shin & Jung, 2019). However, some circumstances such as living alone have been shown to be predictors of lower relational but not subjective wellbeing (Mellor et al., 2008). The overall finding is thus that satisfaction with social relationships is related to life satisfaction, but its determinants are not identical to those of life satisfaction. Hence, the relevance of investigating how relational wellbeing relates to work and family trajectories.

Financial wellbeing at older ages has received considerable attention, but studies mostly measure it through objective outcomes (pension or personal or household income around retirement) rather than through the subjective evaluation of the financial situation. The study of the latter allows to investigate how much the perception of one’s own financial situation depends on material circumstances or their subjective evaluation (Easterlin, 2006). More importantly, financial wellbeing has rarely been studied in relation to both employment and family trajectories despite the recognized importance in life course studies of both domains especially for women’s later wellbeing (Madero Cabib and Fasang, 2016) and, due to the lack of appropriate biographical data, researchers mostly use cross-sectional work and family events, or summary indicators of much more complex life course histories (Halpern-Manners et al., 2015).

### The Swiss Context

Switzerland is classified as a conservative–liberal welfare state with strong traditionalist elements, historically modest universal transfers and a high degree of dependence on labour income (Esping-Andersen, 1990). From the seventies onwards, there has been a large increase in part-time work, with women strongly over-represented in it (Widmer & Ritschard, 2009; Widmer et al., 2003). In 2019, the female part-time employment rate was 61.7% compared to a European average of 29.9% (Eurostat), while 17.1% of Swiss employed men work part-time (8.4% in the EU). Compared with other Western countries, the Swiss unemployment rate has always been extremely low, below 2% until the early nineties and below 5% afterwards (OECD), although long-term unemployment exceeds the OECD mean (Lalive & Lehmann, 2017). Women are more likely to be unemployed (5.1% compared to 4.3% for men in 2018) and more likely than men to be out of the labour force at least for some part of the life course (19.8% of Swiss women were inactive in 2019 versus 11.7% of Swiss men), although both gaps have narrowed in the last decades (Lalive & Lehmann, 2017) and overall female labour force participation is high in international comparison (82.8% in Switzerland versus 71.1 in the EU).

Switzerland’s incentives for a traditional male breadwinner–female caretaker division include gender-segregated labour markets, high gender employment as well as wage gaps; generous-dependent tax allowances, household instead of individual taxation and high marginal tax rates that penalize second earners (Cooke & Baxter, 2010). Furthermore, limited and expensive public childcare and the high costs of existing services equally set strong trade-offs between employment and care time for mothers (Wall & Escobedo, 2013). Swiss women on average undertake 64% (66% among mothers) of housework tasks (Nollert & Gasser, 2017), which is more in line with Southern (Italy 70.1%; Spain 66.5%) than Continental (France 62%; Germany 61.6%) European countries (OECD 2020). Overall, Switzerland displays great gender divides in family responsibilities that relapse almost entirely on women, who end up with a weaker and irregular labour market attachment over the life course. Women still significantly reduce their participation on the labour market during the transition to parenthood and often do not return to full-time work afterwards (Widmer et al., 2003). As a consequence, while men maintained fairly stable and linear occupational trajectories throughout the birth cohorts of the first half of the twentieth century, women’s occupational trajectories display much greater diversification (Widmer & Ritschard, 2009).

### Research Questions and Hypotheses

The aim of the current study is to investigate whether early to mid-adulthood professional and family trajectories (age 20–50) *jointly* affect well being later on. First, *we hypothesize not only that both domains influence wellbeing, but also that family and work histories interact in shaping wellbeing later in life (H1)*. Second, in line with what the life course cumulative disadvantage literature predicts, *we expect that accounting for social origin weakens (but not entirely explains) the association between work and family trajectories and wellbeing (H2)* by influencing the likelihood of experiencing a certain work–family trajectory in the first place. Third, we argue that family and work trajectories, and later wellbeing are *directly associated, beyond the indirect association they have through the family and employment status respondents hold when wellbeing is measured (H3)*.

In relation to gender differences in the association between family and work trajectories and wellbeing, given the more difficult reconciliation between the two spheres and the greater complexity of women’s life courses in Switzerland illustrated above, we expect a *stronger association and a stronger interaction between trajectories and wellbeing for women than for men (H4)*.

## Data and Method

### Data and Sample Selection

We draw on data from the first 19 waves of the large-scale, nationally representative longitudinal Swiss Household Panel (SHP, 1999–2017). The study annually surveys all members (14 and older) of a random sample of private households in Switzerland. Two subsamples of the SHP completed biographical retrospective calendars providing entire work and family histories, in 2002 (*N* = 5560) and 2013 (*N* = 6090). We focus on life course trajectories during prime working and childbearing age. We select respondents who provided complete family and work trajectories covering every year for the ages of 20–50 either in 2002 or 2013 (*N* = 3087, *T* = 31). To obtain wellbeing measures, we select respondents who participated in at least one wave following the collection of the biographical data (2003–06 and 2014–17, respectively).^1^ As the age of respondents filling in the biographical calendar in 2002 and 2013 varies, the age at which wellbeing is measured potentially lies between 51 and 93 years old. To increase the homogeneity of the sample, we restrict it to respondents whose wellbeing is measured between 51 and 70 years old (*N* = 2302). After excluding missing data^2^ on control variables, our final analytical sample consists of 1885 individuals (*N* = 1005 from women and *N* = 880 for men), with retrospective information covering 31 years.

### Variables

Based on the biographical information, we construct the prime working and childbearing age partnership, childbearing and employment trajectories. We based the construction of sequences on the following states in the family sphere: being unpartnered, partnered or re-partnered after union termination (dissolution or widowhood) in combination with being childless or a parent; and the following employment states: being in education, in full-time, large (50–89%) or small (< 50%) part-time employment and non-employment. Being unemployed is a rare event in our sample, and hence, we could not distinguish it from inactivity. For the same reason of a small number of observations, we did not distinguish divorce, separation and widowhood. Appendix Table 4 illustrates the distribution of family and employment states by gender.

The SHP provides an extensive list of indicators of wellbeing recorded in the yearly waves. We focus on general life satisfaction, satisfaction with personal relationships and satisfaction with the financial situation.^3^ All satisfaction indicators are measured on a scale from 0 (not at all satisfied) to 10 (completely satisfied). In all models, we control for age^4^ (51–70) and the period in which wellbeing is measured (2014–17 vs 2003–06). Appendix Table 5 reports summary statistics of the dependent and independent variables included in the analysis.

To test whether the association between specific family and work trajectories and wellbeing exists beyond the selection process into certain types of trajectories, we control for a number of background characteristics, all measured prior to the starting age range of the trajectories (before age 20). The survey includes socio-demographic and social origin information such as country of birth and nationality, living arrangement at age 15 and fathers’ educational level.

Reverse causality between wellbeing and life course trajectories represents a potential bias of our estimates. Happier individuals might experience more positive family and work histories. The association between certain trajectories and wellbeing might be explained by innate conditions that make some individuals happier than others and also more likely to experience a given trajectory. Unfortunately, we do not dispose of information on pre-trajectory wellbeing, but we do have information on physical and mental health problems before age 20 from the health calendar collected in 2013. We use this information as an (imperfect) proxy for subjective wellbeing. Since this would greatly reduce our sample size, we did not include it in the main analyses, but we conducted robustness checks on the 2013 sample, controlling for early life health indicators. Results for life satisfaction are presented in Appendix.

Finally, to investigate how much of the association between family and work trajectories and wellbeing is mediated by the conditions at the time of the survey, we add current marital status (unpartnered; married or registered partnership; and divorced, separated or widow), whether men have had children since^5^ employment status (full-time work; part-time work; inactive; and unemployed), presence of current health problems and net personal income (only in financial wellbeing models). All these variables are measured at the same time as wellbeing.

### Method

Among the studies that investigate both domains together and their joint influence on wellbeing, the majority does not model explicitly the domain interaction (Lippert & Damaske, 2019). In particular, less is known regarding how the two life domains’ long-term trajectories interplay in affecting wellbeing (Aisenbrey & Fasang, 2017). Halpern-Manners et al., (2015) show that trajectory measures predict outcomes better than using point and summary measures (such as the number of events) because they better capture the full temporal dimension of life course pathways. Here, we utilize sequence analysis to identify and describe the different trajectories defined by labour market and family transitions. Sequence analysis offers advantages in terms of investigating the life course in a dynamic longitudinal perspective, distinguishing the unfolding of trajectories from earlier experiences and stable factors like social background and preferences. At the same time, results from sequence analysis make these complex and heterogeneous life courses much easier to interpret (Aassve, Billari et al., 2007).

For the sequence analysis, to compare the trajectories and form the typical clusters, we use dynamic Hamming distance, hierarchical clustering and Wards linkage to identify the family and work clusters separately.^6^ Clustering allows us to identify groups of individuals displaying similar family and work histories. We allow different clustering for men and women as, first, the trajectories likely differ and, second, the complexity might differ across gender. (For instance, Swiss women’s work histories might be more complex than Swiss men’s.) The choice of the number of clusters was based on theoretical grounds and multiple quality criteria (see Appendix Table 6 for size, R-squared, average Silhouette width and Calinski–Harabasz index). The quality criteria do not uniquely indicate a solution, as expected, and we additionally need to balance a sufficient sample size of groups to be interacted across domains with a variety of trajectories as rich as possible. The three groups clustering seems to be the most homogeneously supported solution across quality measures, sample size and maximum variation. A more detailed description of the clusters is presented in the next section.

Once the clusters of typical trajectories are identified, they are treated as categorical explanatory variables. Linear OLS regression models assess the association between typologies of family and work trajectories, their interaction and the wellbeing outcomes.^7^ We opt for constructing the trajectories separately for the two domains and interact the derived clusters instead of using multichannel sequence analyses because “the joint typologies cannot be regarded as proof of a relationship” (Piccarretta and Studer 2019: pp. 6). Conclusions based on multichannel analysis can be drawn only on the mutual association between the domains and not on the possible dependence of the trajectory in one domain on the trajectory in the other domain. In other words, this approach is more complete and flexible since all possible combinations between trajectories in the two domains are considered, not only those produced by the multichannel analysis. Finally, the results of the clustering based on one domain only are easier to interpret.

We test our first two hypotheses (*H1–H2*) of an association between trajectories and wellbeing and its persistence net of pre-trajectory resources by comparing two models: Gross and Net, where in the former we only control for age and period, while in the second we add the pre-trajectories determinants. We test our third hypothesis (*H3*) of the existence of both a direct and an indirect association between trajectories and wellbeing by further adding current family, employment, health and income status. Given the highly gendered family and employment regimes in Switzerland, we not only allow for different clustering, but we test the extent to which the associations between trajectories and wellbeing are gendered (H4), running separate models for men and women.

To favour an easier interpretation, in the next sections, results are presented graphically. Tables with complete models are available in Appendix of the paper.

## Family and Employment Trajectories

**Fig. 1:**
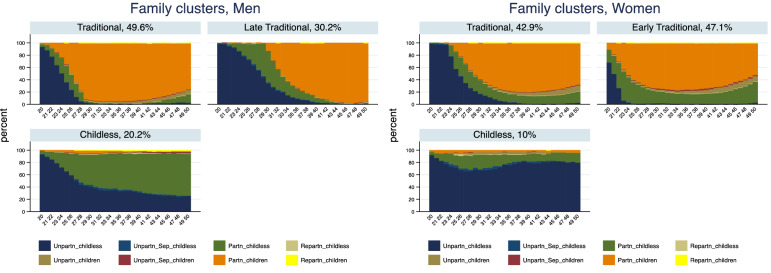
State distribution plots of the family clusters. *Source*: Elaboration of the authors based on SHP Biographical files 2002, 2013

**Fig. 2:**
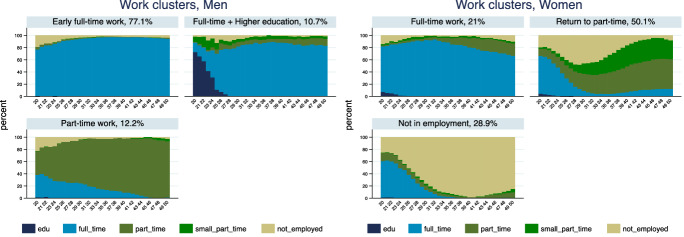
State distribution plots of the employment clusters. *Source*: Elaboration of the authors based on SHP Biographical files 2002, 2013

Figures 1, 2 display the state distribution plots of family and work states by the clusters of typical trajectories identified for men and women. State distribution plots (Billari & Piccarreta, 2005) aggregate the frequency of each state at each time point; therefore, they give a good overview of the time point–specific distribution of states, yet do not display individual sequences. We identified three clusters for men’s typical family trajectories (Fig. 1). Half of Swiss men cluster in a traditional family trajectory group with a relatively early transition into a partnership and fatherhood around their early to mid-twenties (“Traditional”). One-third of men group into a late traditional cluster in which these transitions take place a little later, around the age of 30 (“Late Traditional”). The state plot shows that in these two clusters after the age of 30, the majority of men remain partnered with children. In the late traditional cluster, between the age of 20 and the early 30 s there is still a predominance of childlessness among men. The last cluster (“Childless”, 20%) groups men who mostly remain childless for the entire age interval observed. Panel (a), Table 1 reports the distribution of states within typical trajectories showing that the most prevalent family states in the traditional trajectories for men are partnered with children, while the most prevalent in the childless trajectories are unpartnered and partnered childless.

**Table 1:** States distribution within typical family and work trajectories. Men and women. *Source*: Elaboration of the authors based on SHP Biographical files 2002, 2013

*(a)*					
Family states	Men’s family trajectories				
		Traditional	Late traditional	Childless	Total
Unpartnered, childless	N	1908	2591	2569	7068
	*%*	*14.94*	29.74	44.32	25.91
Unpartnered separated/div/widow, childless	N	66	74	96	236
	*%*	*0.52*	0.85	1.66	0.87
Partnered, childless	N	966	1452	2800	5218
	*%*	*7.56*	16.67	*48.30*	19.13
Re-partnered, separated/div/widow, childless	N	12	16	60	88
	*%*	0.09	0.18	*1.04*	0.32
Unpartnered, with children	N	361	39	3	403
	*%*	2.83	0.45	0.05	1.48
Unpartnered separated/div/widow, with children	N	78	6	54	*138*
	*%*	*0.61*	*0.07*	*0.93*	0.51
Partnered, with children	N	9306	4523	142	13,971
	*%*	72.86	51.92	*2.45*	51.21
Re-partnered, separated/div/widow, with children	N	75	10	73	158
	*%*	*0.59*	0.11	*1.26*	*0.58*
Total	N	12,772	8711	5797	*27,280*

Family states	Women’s family trajectories				
		Traditional	Early traditional	Childless	Total
Unpartnered, childless	N	2955	761	2323	6039
	*%*	*23.36*	*4.92*	*76.46*	*19.38*
Unpartnered separated/div/widow, childless	N	112	117	77	306
	*%*	*0.89*	*0.76*	*2.53*	*0.98*
Partnered, childless	N	2068	3583	499	6150
	*%*	*16.35*	*23.16*	*16.43*	*19.74*
Re-partnered, separated/div/widow, childless	N	46	58	8	112
	*%*	*0.36*	*0.37*	*0.26*	*0.36*
Unpartnered, with children	N	446	721	54	1221
	*%*	*3.53*	*4.66*	*1.78*	*3.92*
Unpartnered separated/div/widow, with children	N	74	247	4	325
	*%*	*0.59*	*1.60*	*0.13*	*1.04*
Partnered, with children	N	6922	9899	73	16,894
	*%*	*54.73*	*63.99*	*2.40*	*54.23*
Re-partnered, separated/div/widow, with children	N	25	83	0	108
	*%*	*0.20*	*0.54*	*0.00*	*0.35*
Total	N	12,648	15,469	3038	31,155

*(b)*					
Work states	Men’s work trajectories				
		Early full-time	Full-time + High edu	Part-time work	Total
In education	N	2	304	4	310
	*%*	*0.01*	*10.01*	*0.12*	*1.14*
Full-time	N	19,491	2249	648	22,388
	*%*	*93.29*	*74.03*	*19.35*	*82.07*
Part-time 50–89%	N	325	246	2466	3037
	*%*	*1.56*	*8.10*	*73.66*	*11.13*
Small part-time < 50%	N	18	133	40	191
	*%*	*0.09*	*4.38*	*1.19*	*0.70*
Not employed	N	1058	106	190	1354
	*%*	*5.06*	*3.49*	*5.68*	*4.96*
Total	N	20,894	3038	3348	27,280

Work states	Women’s work trajectories				
		Full-time work	Return to part-time	Not in employment	Total
In education	N	57	72	15	144
	*%*	*0.86*	*0.43*	*0.19*	*0.46*
Full-time	N	5455	3116	1252	9823
	*%*	*82.23*	*18.55*	*16.22*	*31.53*
Part-time 50–89%	N	686	5807	604	7097
	*%*	*10.34*	*34.56*	*7.82*	*22.78*
Small part-time < 50%	N	142	3773	170	4085
	*%*	*2.14*	*22.46*	*2.20*	*13.11*
Not employed	N	294	4034	5678	10,006
	*%*	*4.43*	*24.01*	*73.56*	*32.12*
Total	N	6634	16,802	7719	31,155

Women’s family clusters differ with respect to, first, the age at family formation, which is lower compared to men. In the biggest cluster, including 47% of women (“Early Traditional”), already at the age of 20, many of them are partnered and some of them have children. In their mid-twenties, more than half of women in this cluster have children. The second cluster with around 43% of women (“Traditional”) still displays a traditional transition to partnership and motherhood, but a bit later compared with the early transition group. Here, women tend to have children around their late twenties. Notably, both clusters, as shown in Fig. 1 and Table 1, include some separation and re-partnering for Swiss women during the last ten years of the life course trajectory considered, which we did not observe for men with the same intensity. Yet, those states are not frequent enough to constitute a separate cluster. The third cluster includes 10% of women (“Childless”) and, as Fig. 1 illustrates, across all ages the most prevalent state is the one of being unpartnered and childless. Therefore, the second difference between Swiss men and women regarding typical family trajectories is that while the cluster of childless men equally include partnered (48.3% of states, Table 1) and unpartnered (44.3% of states, Table 1) men, women in the childless cluster are predominantly unpartnered (76.5% of states, Table 1). Based on the previous studies illustrated earlier, we can hypothesize that this cluster of women would be more disadvantaged in terms of wellbeing compared to men since besides kids, they also tend to lack a stable relationship.

Table 2 reports the educational-level distribution in each cluster^8^.^,^ 9 While around 70% and 67% of women in the early traditional and traditional clusters, respectively, have an upper secondary education at most, this proportion is 52% in the cluster of childless women, who are more likely to have a tertiary degree than women with a family. For men, educational differences across family clusters are much smaller, although the highest proportion of tertiary educated men is found in the traditional late group (53%) and not among the childless men who actually display the lowest proportion of tertiary educated among the three clusters.

**Table 2:** Educational-level distribution by family and employment clusters. Men and women. *Source*: Elaboration of the authors based on SHP Biographical files 2002, 2013. Row percentages

Women's family clusters	Primary (%)	Upper secondary (%)	Tertiary (%)	Total (*N*)
Traditional	7.35	66.67	25.98	408
Early traditional	14.03	70.54	15.43	499
Childless	14.29	52.04	33.67	98
Total	11.34	67.16	21.49	1005

Men's family clusters	Primary (%)	Upper secondary (%)	Tertiary (%)	Total (*N*)
Traditional	3.88	53.64	42.48	412
Late traditional	3.56	43.42	53.02	281
Childless	2.67	55.61	41.71	187
Total	3.52	50.8	45.68	880

Women's work clusters	Primary (%)	Upper secondary (%)	Tertiary (%)	Total (*N*)
Full-time work	12.15	57.94	29.91	214
Return to part-time work	9.41	68.08	22.51	542
Not in employment	14.86	73.09	12.05	249
Total	11.34	67.16	21.49	1′005

Men's work clusters	Primary (%)	Upper secondary (%)	Tertiary (%)	Total (*N*)
Early full-time work	3.41	58.16	38.43	674
Full-time work after higher education	1.01	4.08	94.9	98
Part-time work	6.48	47.22	46.3	108
Total	3.52	50.8	45.68	880

Figure 2 shows the state distribution plots for the work domain by the identified typical clusters. Swiss men disproportionately work full-time during their employment trajectories. The vast majority of them enter the labour market quite early, as in the case of the first cluster comprising around 77% of men in the sample, and stay in full-time employment for their prime-working age (“Early full-time work”). The state plot and Panel (b) in Table 1 show that men in this group rarely experience joblessness and especially at the beginning of their career and work very little part-time. The second largest cluster (“Part-time work”, 12%) include men who mostly work in a 50–89% part-time job. Some of these men work full-time when they enter the labour market, but part-time work heavily prevails in most of their career. Finally, the last cluster of men of similar size (“Full-time work after higher education”, 11%) resembles the first regarding the predominance of full-time work; however, in this cluster men stay longer in education and enter the labour market a bit later. As Fig. 2 shows by the age of 23–24, still 40% of them are in education and, in fact, 95% of them are tertiary educated compared to the 38% of those in the early labour market entry cluster and 46% of the part-time work cluster (Table 2).

Women’s employment trajectories in Switzerland are very different from men. Almost one-third of them cluster into the group of the not employed for most of their prime working age (“Not in employment”). Almost 80% of them work full- or part-time early in the career, but by the age of 30, this share is below 20% (Fig. 2 and Table 1). The largest cluster of women (“Return to part-time work”, 50%) is mostly characterized by a similar labour supply decline during childbearing years, between the mid-twenties and the mid-thirties, but also by a return to the labour market working part-time. Figure 2 shows that in this cluster by the age of 40, more than 80% are employed again. Finally, 21% of women in the sample cluster in the full-time work group (“Full-time work”). In this cluster, non-employment is rare and concentrated very early or late in the career, and although by the age of 50 almost a quarter of them works part-time, most of their prime working age is spent in full-time work. There is quite a large difference in the educational level of women in the three clusters. Unsurprisingly, the largest share of women with tertiary education is found in the full-time cluster where 30% of women have university education. In the return to part-time cluster, 22.5% of women have tertiary education, while only 12% do in the not-employed cluster. These descriptive statistics suggest that disadvantages tend to accumulate and less skilled workers, especially women, tend to have a weaker attachment to the labour market than highly skilled ones.

Table 3 presents the joint distribution of the clusters in the sample. The largest group of men in the sample (38%) belongs to the early full-time job trajectory in combination with the traditional family trajectory, while the second and third largest groups of men (23.2% and 15.5%, respectively) belong to the same early full-time job trajectory but in combination with a later family formation or childless trajectory. The rarest combination comprises the childless trajectory with full-time job with higher education (below 2% in the sample). Among Swiss women, the most common combinations are an early (31%), or a slightly postponed family formation (21%) together with a return to part-time work after childrearing age. The subsequent most common clusters combine joblessness with family formation, while childlessness is in general quite infrequent (3–7%), and extremely rare in combination with the employment trajectory of non-employment (0.4%).

**Table 3:** Joint distribution of family and employment clusters. Men and women. *Source*: Elaboration of the authors based on SHP Biographical files 2002, 2013. Cell percentages

Men	Traditional	Late traditional	Childless	Total (*N*)
Early full-time work	37.95	23.18	15.45	674
Full-time work after higher education	4.2	5.11	1.82	98
Part-time work	4.66	3.64	3.98	108
Total (N)	412	281	187	880

Women	Traditional	Early traditional	Childless	Total (*N*)
Full-time work	6.47	7.96	6.87	214
Return to part-time	20.8	30.65	2.49	542
Not in employment	13.33	11.04	0.4	249
Total (N)	408	499	98	1′005

## Multivariate Analysis Results

**Fig. 3:**
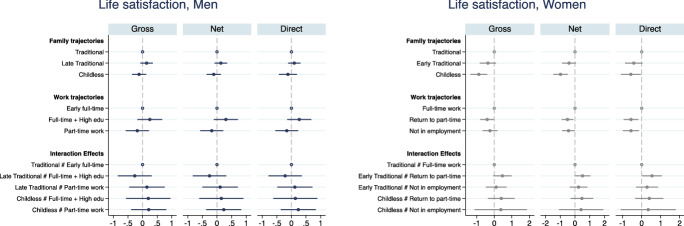
OLS estimates of family and work trajectories’ association with life satisfaction. Interaction model. Men and women. *Source*: Elaboration of the authors based on SHP Biographical files 2002, 2013 and SHP panel (2003–2017). *Note* Gross model controls only for age and period; Net model controls for age, period and pre-trajectory controls; Direct model controls for age, period, pre-trajectory controls and current controls

**Fig. 4:**
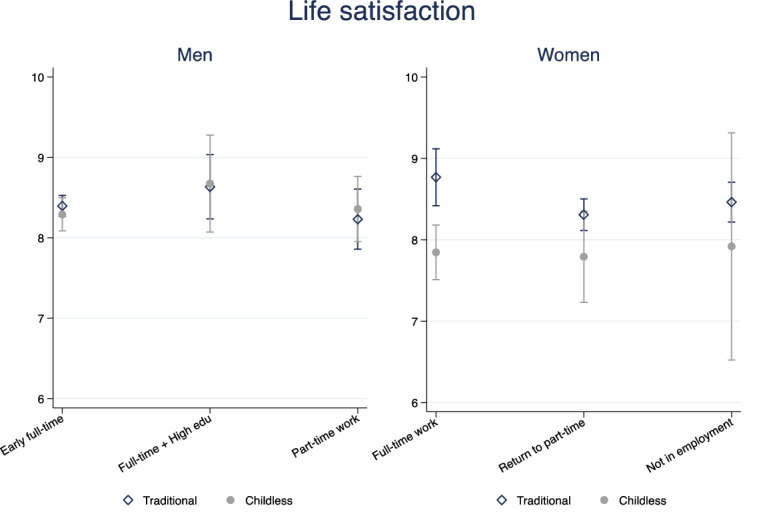
Predicted life satisfaction, interaction model. Men and women. *Source*: Elaboration of the authors based on SHP Biographical files 2002, 2013 and SHP panel (2003–2017)

**Fig. 5:**
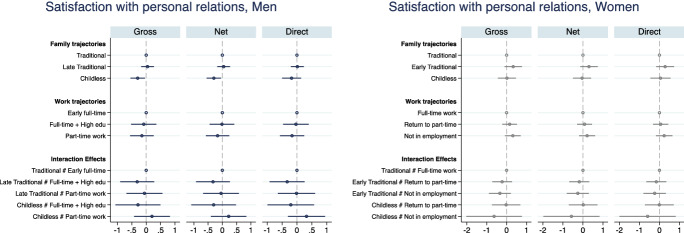
OLS estimates of family and work trajectories’ association with satisfaction with personal relationships. Interaction model. Men and women. *Source*: Elaboration of the authors based on SHP Biographical files 2002, 2013 and SHP panel (2003–2017). *Note* Gross model controls only for age and period; Net model controls for age, period and pre-trajectory controls; Direct model controls for age, period, pre-trajectory controls and current controls

**Fig. 6:**
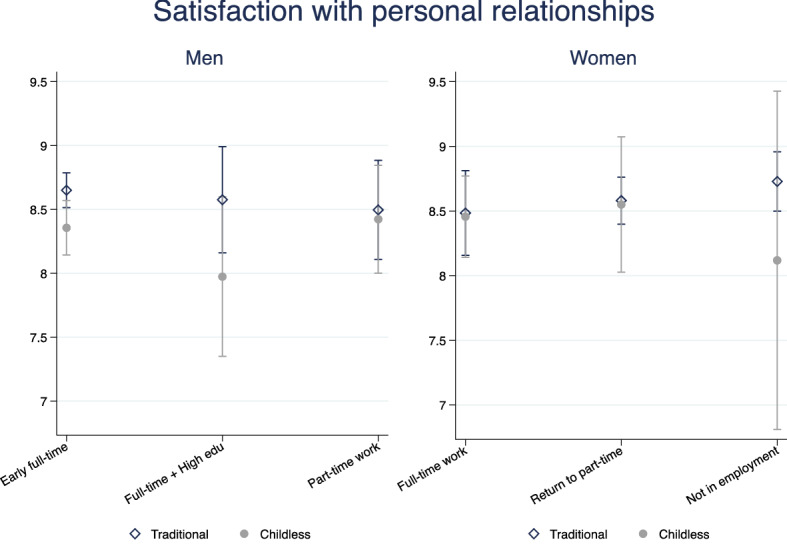
Predicted satisfaction with personal relationships, interaction model. Men and women. *Source*: Elaboration of the authors based on SHP Biographical files 2002, 2013 and SHP panel (2003–2017)

**Fig. 7:**
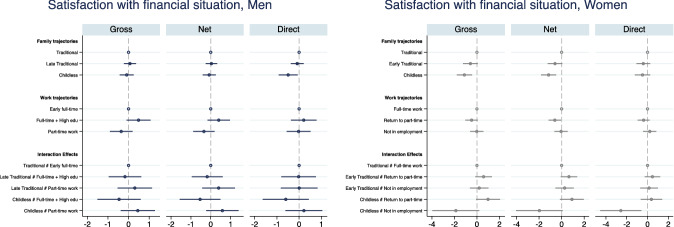
OLS estimates of family and work trajectories’ association with satisfaction with financial situation. Interaction model. Men and women. *Source*: Elaboration of the authors based on SHP Biographical files 2002, 2013 and SHP panel (2003–2017). *Note* Gross model controls only for age and period; Net model controls for age, period and pre-trajectory controls; Direct model controls for age, period, pre-trajectory controls and current controls

**Fig. 8:**
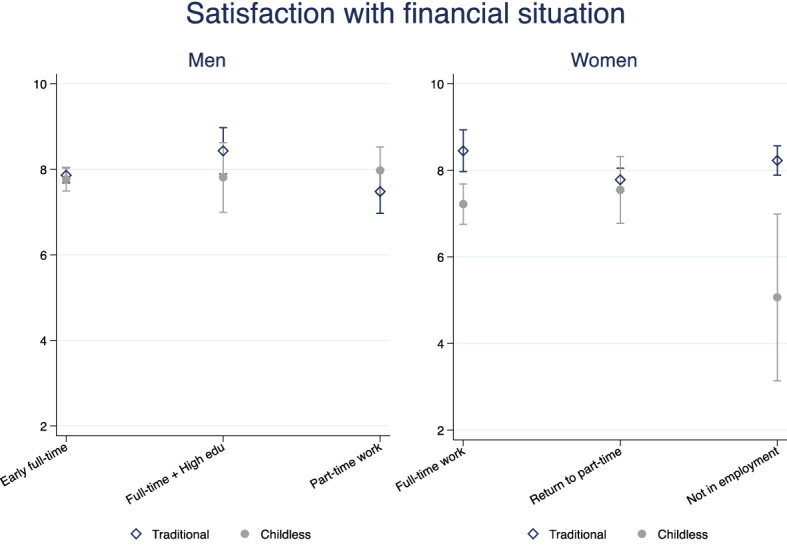
Predicted satisfaction with financial situation, interaction model. Men and women. *Source*: Elaboration of the authors based on SHP Biographical files 2002, 2013 and SHP panel (2003–2017)

Figures 3, 4, 5, 6, 7, 8 present the results from the OLS linear regression models for three wellbeing outcomes: life satisfaction, satisfaction with personal relationships and with the financial situation. All figures present on the left panel results for men and on the right panel results for women. Complete tables are included in Appendix (Tables 7,8,9,10,11,12).

Figure 3 plots the coefficients of the association between family and work trajectories, and their interaction, and subjective wellbeing from the three different models (gross, net of pre-trajectory determinants, and direct, controlling for current status). For men, family trajectories are not significantly associated with subjective wellbeing in themselves nor in interaction with any type of employment trajectory. Table 13 in Appendix shows that controlling for having experienced health issues before the age of 20, the combination of a late family formation with early full-time work is associated with higher life satisfaction for men. This might also be a result of the younger cohorts included in the sample for these robustness checks analyses, but it seems that securing a stable career before starting a family is for Swiss men associated with higher subjective wellbeing later on.

For working women without a family (the cluster Childless), life satisfaction is substantially lower compared with working women who have children during their life course. The association is not explained by pre-trajectory resources (not even by health conditions before the age of 20, Appendix Table 13), and part of the association is direct: Once controlling for the proximate determinants (Appendix Table 8), the negative direct relationship between childlessness and women’s subjective wellbeing declines but remains negative and significant. This is not so surprising, as women who have been childless for most of their lives are likely to remain in this status after the age of 50, when wellbeing is measured. Current partnership status is strongly associated with men and women subjective wellbeing but differently, as previous studies suggest. While being single instead of partnered reduces life satisfaction among both men and women, being separated, divorces or widow is associated with a lower life satisfaction only among women.

Women’s weaker attachment to the labour market—both as non-employment or later return to part-time—in combination with a late traditional family formation, is also significantly associated with lower life satisfaction net of resources and in a direct as well as indirect manner. While early family formation and the return to work after childrearing are each negatively associated with wellbeing relative to late family and full-time work, their combination appears to attenuate the lower life satisfaction. The earlier women have children, the earlier they re-enter the labour market (provided that they re-enter) and the longer they profit from the beneficial effects of being employed.

To give a more exhaustive picture of the wellbeing profiles associated with given family and work trajectories, Fig. 4 presents predicted life satisfaction^10^ for relevant clusters of family and work trajectories.^11^ Among women with a traditional family trajectory, those with a full-time work trajectory score significantly higher on life satisfaction (around 0.5 points in the 0–10 scale) compared with those who experience a break in their career (not statistically significant for the not employed trajectory). Within the group of women with a full-time working trajectory, those with a traditional family trajectory score about 1 point higher on life satisfaction than childless women. The latter display a pretty stable subjective wellbeing irrespectively of their working histories; even a trajectory of stable full-time work does not compensate for the lower subjective wellbeing of women without children. It is full-time working women with a traditional family history that have an advantage with respect to all other family–work constellations. Career interruptions during childrearing do not pay off for women later in life. For Swiss men, instead, looking at predicted life satisfaction (Fig. 4) across family and work trajectories we find no statistically significant differences (identical results are obtained looking at traditional late family formation—not shown).

Figures 5, 6 report results on relational wellbeing. For men with a weak attachment to the labour market, we do not observe any difference across family trajectories, but for men in the early full-time work trajectory, we find that having a family with children is associated with higher relational wellbeing compared with remaining childless (Fig. 6). The association is not explained by early resources that drive men into long-term childlessness; however, as soon as they have a child later on, the association disappears (Fig. 5). It is interesting to note that among men, being currently unpartnered rather than married or in a registered partnership is associated with a 1-point drop in satisfaction with relationships, relative to a baseline satisfaction of 7.7 (Appendix Table 9). This is not observed among women whose relational wellbeing seems to be unrelated to current marital status and their family history. This is in line with previous literature showing that women more successfully substitute the missing support of a partner, fostering larger networks of friends and family (Baumbusch, 2004; Zimmermann & Hameister, 2019).

Early, compared to later family formation appears to be associated with higher satisfaction with personal relationship, but the difference is not statistically significant (Fig. 5). No association is visible between employment trajectories and satisfaction with personal relations among women. The strongest determinant of women’s relational wellbeing at older age is their living arrangement when adolescent: growing up with a lone parent reduces women’s satisfaction with personal relationships by 0.3 points (Appendix Table 10). Overall, it seems that relational wellbeing is linked more to family ties than labour market ties for both Swiss men and women. However, the association with long-term family and employment trajectories is very weak and social origin (for women) and current family status (for men) are the strongest determinants of relational wellbeing.

We do not find that family and work trajectories interact in affecting men’s financial wellbeing in Switzerland (Figs. 7, 8). Given the low variation in Swiss men’s trajectories, the general stability of male’s professional lives over the life course and the implied financial security, this result is not surprising. Swiss women’s trajectories of family and professional life are more heterogeneous than men’s, and these complexities are likely to be problematic for women’s financial wellbeing. Figures 7, 8 show that long-term childlessness (mostly coupled with singlehood) is associated with a significantly lower satisfaction with the financial situation compared with women who do have a family. This is a disadvantage that is independent of women’s labour market history. Moreover, the negative association between having no partner or children and financial wellbeing is not explained by women of low social origin or pre-trajectory conditions, but it disappears once the current situation is taken into account (Fig. 7). It is the lower income of single women that explains the negative effect of not having had a partner (and/or children) throughout most of their lives on financial wellbeing (Fig. 7). The risk of lower financial wellbeing for women with no families is common to all types of work trajectories, but the difference with women with a family is smallest and not statistically different from zero among women with a part-time work career. The difference among full-time working women is larger because the financial security of dual-earner couples is higher. (The point estimates gap with childless women is above 1 point in the 0–10 scale, Fig. 8.) Predictably, we find the largest difference among women with non-working trajectories. Women who never worked but do have a partner and a family report a financial wellbeing almost identical to full-time working women in dual-earner couples. Instead, women who never worked and remained childless and often unpartnered most their lives report a significantly lower financial wellbeing. The gap is larger than among working women (although confidence intervals are much larger too) as the point estimates indicate a predicted satisfaction with financial situation of around 5 for the latter group and above 8 for women with a traditional family biography. Finally, the combination of a history of non-employment with no family is persistently associated with lower financial wellbeing of women, even if they re-partner, find a job or their income increases (direct model, Fig. 7). There is a clear long-term risk of much lower financial wellbeing for women who combine a very weak labour market attachment with remaining unpartnered and childless.

## Discussion

The findings of this study are multiple. First, work and family trajectories in prime working age do interplay in determining wellbeing outcomes at later ages (H1). Men’s subjective wellbeing benefits from a delayed entry into the labour market combined with a traditional family formation and from securing a stable career before starting a family (in the youngest 2013 sample). Women enjoy a long-term financial and overall wellbeing advantage when full-time work is combined with a traditional (but not too early) family formation. This confirms earlier studies indicating that a stable attachment to both work and family comes with an economic and mental health premium for both men and women. Satisfaction with personal relationships, instead, showed surprisingly little association with work and family trajectories. Relational wellbeing in Switzerland is strongly linked to current partnership for men, and bonds from family of origin for women.

Second, we find an association between trajectories and wellbeing net of early life resources, such as social origin and socio-economic background (H2). Third, women who during most of their life remain childless or unpartnered, compared to women who form a family, display a lower life satisfaction that remains such even when they partner. Similar results apply to their satisfaction with the financial situation. This suggests a long-lasting effect of women’s weak family trajectories on their financial and overall wellbeing. Our hypothesis that there exists a direct link between trajectories and wellbeing is thus supported (H3).

All in all, Swiss women’s wellbeing at later ages is more than men’s dependent on family and work trajectories, and their interaction (H4). While family ties are paramount for women’s overall and financial long-term wellbeing, the beneficial effect of family history is moderated by professional ties. Women who are unpartnered and childless for most their lives report a particularly lower financial wellbeing if they never worked. However, women who never worked but have had a family report a financial wellbeing almost identical to full-time working women in dual-earner couples. Our findings are in line with previous studies showing that working partnered mothers in Switzerland display the highest and single women the lowest wellbeing (Perrig-Chiello et al., 2008). However, our findings further suggest a cumulative long-term risk of low financial wellbeing for women who combine a very weak labour market attachment with no family formation. Moreover, despite employment attenuating this vulnerability, even full-time work does not compensate entirely for the financial dependence on a more normative family form.

The study has a few limitations. First, physical and mental health problems before age 20 provide only an imperfect proxy of pre-trajectory wellbeing. Reverse causality between wellbeing and life course trajectories hence still represents a potential bias of our estimates. The association we observe between certain trajectories and wellbeing might be explained by innate conditions that make some individuals happier and more likely to experience a given trajectory. However, while physical and mental health alone might not provide an exact measure of innate wellbeing, we are confident that that coupled with the rich array of other pre-trajectory indicators, we include (social origin, parental social status and living conditions during adolescence) very closely picture the wellbeing conditions that might lead to more or less privileged trajectories.

Second, the rarity of some of the most vulnerable trajectories in Switzerland hinders a sharp distinction between the representative trajectories. For instance, the most insecure work trajectory among men is that of part-time work, which may reflect underemployment but may also result from men choosing to dedicate their time to other wellbeing enhancing activities (e.g. leisure, social relationships). Another example is that of family trajectories characterized by multiple marriages or lone parenthood that do not emerge as typical trajectories. Although cases exist, they are not enough to constitute a trajectory on their own. Much of the difference across family trajectories, instead, emerges regarding the age at family formation, which does not seem to make a remarkable difference for long-term wellbeing in the Swiss context. However, even in this relatively protected environment we do spot alarming differences between more and less vulnerable groups.

Third, given the limited number of observations it was not possible to increase the number of critical events used to generate the sequences. Therefore, unemployment could not be distinguished from inactivity which, especially for women, represent very different sources of vulnerability. For the same reason, different types of unions such as marriages and cohabitations, and union dissolutions, such as divorces, separations and widowhood could not be distinguished. Relatedly, due to the reduced sample size, the study could not further address the moderating role of resources in the link between trajectories and wellbeing. Socio-economic and health background characteristics influence not only which family or work trajectories individuals experience, but also how they manage the double commitments to work and family, how they react to critical transitions in life and, therefore, also how wellbeing is affected by those events and trajectories.

Finally, our sequence analysis suffers from limitations that are common to all studies using this method. Being an exploratory data-driven approach, it poses problems with respect to the possibility of handling trajectories only partially observed. The handling of missing data and censored sequences remains an unresolved issue at the moment (Piccarretta & Studer 2019); therefore, as in other studies, we limit the analysis to complete sequences. Creating a missing state for each domain and then interacting them would have created too many categories and uninterpretable estimates. More importantly, the life course holistic interpretation typical of sequence analysis necessarily loses the focus on the theoretical mechanisms behind events and transitions that generate a particular long-term trajectory and of studying the impact of time-varying covariates on life courses. For these reasons, the holistic approach is rather complementary to other model-based analyses of the life course (Piccarreta & Studer, 2019).

Notwithstanding such limitations, this study robustly shows a stronger interaction of family and work trajectories in shaping overall and financial wellbeing in older age for women compared to men. We confirm previous studies (Halpern-Manners et al., 2015; Madero-Cabib & Fasang, 2016) by showing that the spillover between work and family has consequences for women’s wellbeing also beyond childbearing ages, but we also introduce novel perspectives. For instance, Madero-Cabib and Fasang (2016) show that Swiss women who combine early motherhood with a weak attachment to the labour market suffer lower financial wellbeing at retirement age. However, our study shows that this is not always the case. Swiss women actually benefit from an early family formation if they return to work after childrearing, because they return at a relatively younger age compared to women who partner and have children later on (who either never return to work or return to work at an older age). We further show that the same moderating positive consequences of returning to work after an early family formation among women influence not only financial but also, and even more, women’s subjective wellbeing. Finally, we add that while the consequences in terms of financial wellbeing of a combination of early family formation and an intermittent career can be resolved if women’s income recovers later on in the life course, the effects on life satisfaction are much more persistent beyond later family and employment events.

The unique contribution of our study on life course development of wellbeing lies in its comprehensive character. The main conclusion we draw is that a biography characterized by a prolonged lack of partnership and children—representing a non-normative family trajectory in the Swiss context—endangers Swiss women’s financial security more than a history of weak attachment to the labour market. The latter, however, generates significant and persistent negative effects on women’s overall happiness. Interestingly, neither men’s nor women’s work trajectories in Switzerland seem to generate long-term positive social network externalities influencing relational wellbeing in older age. This shows the importance of understanding wellbeing in a multidimensional way as different aspects of it are differently determined by early life resources, family and work trajectories and current events.

## Appendix

See Tables 4, 5, 6, 7, 8, 9, 10, 11, 12 and 13.

**Table 4:** Family and work states distribution by gender. *Source*: Elaboration of the authors based on SHP Biographical files 2002, 2013 and SHP panel (2003–2017)

Family states	Gender			
		Man	Woman	Total
Unpartnered, childless	N	7068	6039	13,107
	%	25.91	19.38	22.43
Unpartnered separated/divorced/widow, childless	N	236	306	542
	%	0.87	0.98	0.93
Partnered, childless	N	5218	6150	11,368
	%	19.13	19.74	19.45
Re-partnered, separated/divorced/widow, childless	N	88	112	200
	%	0.32	0.36	0.34
Unpartnered, with children	N	403	1221	1624
	%	1.48	3.92	2.78
Unpartnered separated/divorced/widow, with children	N	138	325	463
	%	0.51	1.04	0.79
Partnered, with children	N	13,971	16,894	30,865
	%	51.21	54.23	52.82
Re-partnered, separated/divorced/widow, with children	N	158	108	266
	%	0.58	0.35	0.46
Total	N	27,280	31,155	58,435
	%	100.00	100.00	100.00

Work states		Man	Woman	Total
In education	N	310	144	454
	%	1.14	0.46	0.78
Full-time	N	22,388	9823	32,211
	%	82.07	31.53	55.12
Part-time 50–89%	N	3037	7097	10,134
	%	11.13	22.78	17.34
Small part-time < 50%	N	191	4085	4276
	%	0.70	13.11	7.32
Not in employment nor education	N	1354	10,006	11,360
	%	4.96	32.12	19.44
Total	N	27,280	31,155	58,435
	%	100.00	100.00	100.00

**Table 5:** Summary statistics and distribution of categorical variables. *Source*: Elaboration of the authors based on SHP Biographical files 2002, 2013 and SHP panel (2003–2017)

*(a)*					
Continuous variables	*N*	Mean	Std. Dev.	Min	Max
Life satisfaction	1884	8.38	1.36	0	10
Satisfaction with personal relationships	1885	8.61	1.31	0	10
Satisfaction with financial situation	1883	7.88	1.89	0	10
Age	1885	60.07	5.66	51	70
Net income	1684	67,479.07	80,984.67	100	2,560,900
Net income (Thousands)	1684	67.48	80.98	0.1	2560.9

*(b)*			
Categorical variables	Categories	*N*	%
Women work clusters	Full-time work	214	21.29
	Return to part-time	542	53.93
	Not in employment	249	24.78
Men work clusters	Early full-time	674	76.59
	Full-time + High education	98	11.14
	Part-time work	108	12.27
Women family clusters	Early Traditional	499	49.65
	Traditional	408	40.6
	Childless	98	9.75
Men family clusters	Traditional	412	46.82
	Late Traditional	281	31.93
	Childless	187	21.25
Sex	Men	880	46.7
	Women	1005	53.3
Period	2003	870	46.15
	2014	1015	53.85
Swiss	Born in Switzerland or Swiss national	1337	70.93
	Born abroad	548	29.07
Education	Primary	145	7.69
	Upper Secondary	1222	59.52
	Tertiary	618	32.79
Living arrangement at 14	Lived with both parents	1′571	83.34
	Lived with lone parent	183	9.71
	Lived alone or other living arrangement	131	6.95
Current employment status	full-time paid work	478	25.36
	part-time paid work	364	19.31
	inactive	1023	54.27
	unemployed	17	0.9
	other	3	0.16
Current marital status	Unpartnered	130	6.9
	Married or registered partnership	1392	73.85
	Separated, divorced, widow	363	19.26
Child (men)	Have a child at age 50	756	85.9
	No child	124	14.1
Father's education	Primary	617	32.73
	Upper Secondary	887	47.06
	Tertiary	381	20.21
Health problems before age 20	No health issues before age 20	661	35.07
	Health issues before age 20	354	18.78
	Missing	870	46.15

**Table 6:** Cluster solutions quality criteria. *Source*: Elaboration of the authors based on SHP Biographical files 2002, 2013 and SHP panel (2003–2017)

Family clusters men	*N*	R2	ASW	CH	Family clusters women	*N*	R2	ASW	CH
2 clusters	1200	0.285	0.59	598.1	2 clusters	1423	0.299	0.74	674.3
	303		0.47			158		0.42	
3 clusters	746	0.373	0.27	447.0	3 clusters	745	0.464	0.57	681.9
	454		0.32			678		0.23	
	303		0.39			158		0.37	
4 clusters	746	0.421	0.27	363.2	4 clusters	745	0.533	0.48	598.9
	454		0.31			582		0.32	
	97		0.06			96		0.54	
	206		0.70			158		0.26	

Work clusters men	*N*	R2	ASW	CH	Work clusters Women	*N*	R2	ASW	CH
2 clusters	345	0.264	−0.25	539.1	2 clusters	1248	0.189	0.24	367.3
	1158		0.94			333		0.62	
3 clusters	184	0.511	0.46	784.5	3 clusters	455	0.330	0.58	388.3
	161		− 0.04			793		0.00	
	1158		0.92			333		0.59	
4 clusters	184	0.593	0.46	728.4	4 clusters	455	0.411	0.48	367.6
	6		0.7			466		− 0.01	
	155		0.11			327		0.35	
	1158		0.90			333		0.54	

*N* is the number of observations per cluster. R2, Pseudo R^2^, is the share of the discrepancy explained by the clustering solution. ASW, the average Silhouette width, is the coherence of assignments: high coherence indicates high between-group distances and strong within-group homogeneity. CH, Calinski–Harabasz index, is the Pseudo F computed from the distances

**Table 7:** Predicted life satisfaction. Men. *Source*: Elaboration of the authors based on SHP Biographical files 2002, 2013 and SHP panel (2003–2017)

	Model	Model	Model	Model	Model	Model
	(1)	(2)	(3)	(4)	(5)	(6)
	Gross	Net	Direct	Gross	Net	Direct
*Family trajectory: traditional (Ref)*						
Late traditional	0.117	0.104	0.078	0.139	0.126	0.097
	(−0.071–0.306)	(−0.083–0.291)	(−0.108–0.263)	(−0.076–0.354)	(−0.087–0.340)	(−0.115–0.309)
Childless	−0.073	−0.059	−0.076	−0.120	−0.106	−0.107
	(−0.286–0.140)	(−0.270–0.152)	(−0.370–0.217)	(−0.364–0.125)	(−0.348–0.137)	(−0.416–0.203)
*Work trajectory: early full-time work (ref)*						
Full-time work after higher education	0.166	0.153	0.155	0.249	0.238	0.231
	(−0.107–0.440)	(−0.120–0.427)	(−0.116–0.427)	(−0.175–0.673)	(−0.184–0.660)	(−0.188–0.650)
Part-time work	−0.073	−0.060	−0.048	−0.179	−0.165	−0.142
	(−0.326–0.180)	(−0.311–0.191)	(−0.298–0.203)	(−0.578–0.219)	(−0.560–0.231)	(−0.535–0.250)
Late traditional* full-time work after higher education				−0.266	−0.250	−0.221
				(−0.844–0.311)	(−0.823–0.323)	(−0.790–0.348)
Late traditional*part-time work				0.149	0.122	0.109
				(−0.457–0.756)	(−0.483–0.726)	(−0.493–0.711)
Childless*full-time work after higher education				0.198	0.146	0.131
				(−0.562–0.958)	(−0.608–0.900)	(−0.623–0.885)
Childless* part-time work				0.211	0.232	0.220
				(−0.394–0.816)	(−0.368–0.833)	(−0.379–0.819)
Age	0.032***	0.033***	0.033***	0.032***	0.034***	0.033***
	(0.017–0.046)	(0.019–0.048)	(0.014–0.051)	(0.018–0.047)	(0.019–0.048)	(0.015–0.051)
*Year: 2014 (Ref)*						
Year: 2003	−0.195**	−0.151	−0.139	−0.196**	−0.150	−0.138
	(−0.369–−0.021)	(−0.335–0.033)	(−0.347–0.069)	(−0.370 to −0.021)	(−0.334–0.034)	(−0.346–0.071)
*Born in Switzerland or Swiss nationality (Ref)*						
Born abroad		−0.248**	−0.249**		−0.251**	−0.253**
		(−0.442 to −0.055)	(−0.450 to −0.048)		(−0.444 to −0.057)	(−0.455 to −0.052)
*Lived with both parents (Ref)*						
Lived with lone parent		−0.168	−0.145		−0.173	−0.150
		(−0.433–0.096)	(−0.409–0.118)		(−0.439–0.093)	(−0.415–0.115)
Lived alone or other living arrangements		−0.346*	−0.326*		−0.343*	−0.324*
		(−0.695–0.003)	(−0.672–0.021)		(−0.693–0.008)	(−0.672–0.024)
*Father upper secondary education (Ref)*						
Father Primary Education		−0.196**	−0.174*		−0.191**	−0.171*
		(−0.384 to −0.009)	(−0.360–0.013)		(−0.379–−0.003)	(−0.358–0.016)
Father tertiary education		0.108	0.097		0.104	0.092
		(−0.099–0.316)	(−0.110–0.303)		(−0.104–0.312)	(−0.115–0.299)
*Current marital status: married or registered partnership (Ref)*						
Unpartnered			−0.587***			−0.594***
			(−0.998 to −0.176)			(−1.005 to −0.182)
Sep, div, widow			−0.108			−0.111
			(−0.343–0.126)			(−0.346–0.124)
*Currently have children: Yes (Ref)*						
Currently have children: No			0.325*			0.306*
			(−0.032–0.683)			(−0.055–0.667)
*Suffers from current illness: No (Ref)*						
Suffers from current illness: Yes			−0.162			−0.163
			(−0.359–0.035)			(−0.362–0.035)
*Current employment status: Inactive (Ref)*						
Full-time work			0.074			0.077
			(−0.156–0.303)			(−0.154–0.308)
Part-time paid work			−0.142			−0.153
			(−0.445–0.161)			(−0.458–0.152)
Unemployed			−1.161***			−1.118***
			(−1.913 to −0.408)			(−1.875 to −0.360)
Constant	6.573***	6.592***	6.670***	6.545***	6.570***	6.645***
	(5.663–7.483)	(5.684–7.501)	(5.448–7.892)	(5.632–7.458)	(5.658–7.482)	(5.420–7.871)
Observations	879	879	879	879	879	879
*R*-squared	0.041	0.063	0.091	0.043	0.065	0.093

Confidence intervals in parenthesis. **p* < 0.1, ***p* < 0.05, ****p* < 0.01

**Table 8:** Predicted life satisfaction. Women. *Source*: Elaboration of the authors based on SHP Biographical files 2002, 2013 and SHP panel (2003–2017)

	Model	Model	Model	Model	Model	Model
	(1)	(2)	(3)	(4)	(5)	(6)
	Gross	Net	Direct	Gross	Net	Direct
*Family trajectory: Traditional (Ref)*						
Early traditional	−0.072	−0.052	−0.035	−0.363	−0.369	−0.377
	(−0.259–0.115)	(−0.240–0.136)	(−0.220–0.149)	(−0.828–0.102)	(−0.837–0.099)	(−0.838–0.084)
Childless	−0.665***	−0.692***	−0.332	−0.884***	−0.923***	−0.540**
	(−1.004 to −0.326)	(−1.032 to −0.352)	(−0.733–0.069)	(−1.365 to −0.403)	(−1.406 to −0.439)	(−1.060 to −0.020)
*Work trajectory: Full−time work (Ref)*						
Return to part-time	−0.119	−0.164	−0.220*	−0.405**	−0.461**	−0.516**
	(−0.361–0.123)	(−0.409–0.081)	(−0.466–0.026)	(−0.800 to −0.009)	(−0.860 to −0.063)	(−0.910 to −0.123)
Not in employment	−0.146	−0.183	−0.348**	−0.246	−0.307	−0.482**
	(−0.427–0.136)	(−0.467–0.101)	(−0.637 to −0.058)	(−0.671–0.179)	(−0.738–0.125)	(−0.910 to −0.053)
Early traditional*return to part−time				0.463*	0.486*	0.505*
				(−0.064–0.991)	(−0.043–1.015)	(−0.014–1.023)
Early traditional*not in employment				0.109	0.157	0.202
				(−0.479–0.697)	(−0.435–0.748)	(−0.378–0.781)
Childless*return to part-time				0.398	0.407	0.358
				(−0.362–1.159)	(−0.355–1.170)	(−0.384–1.100)
Childless*not in employment				0.371	0.380	0.317
				(−1.121–1.864)	(−1.114–1.874)	(−1.135–1.769)
Age	0.022***	0.022***	0.023**	0.022***	0.023***	0.023**
	(0.006–0.038)	(0.006–0.038)	(0.005–0.041)	(0.006–0.038)	(0.006–0.039)	(0.005–0.042)
*Year: 2014 (Ref)*						
Year: 2003	−0.363***	−0.313***	−0.294***	−0.365***	−0.315***	−0.291***
	(−0.546 to −0.181)	(−0.506 to −0.120)	(−0.499 to −0.089)	(−0.549 to −0.182)	(−0.509 to −0.121)	(−0.497 to −0.086)
*Born in Switzerland or Swiss nationality (Ref)*						
Born abroad		−0.194*	−0.221**		−0.193*	−0.224**
		(−0.395–0.006)	(−0.419 to −0.022)		(−0.395–0.009)	(−0.424 to −0.024)
*Lived with both parents (Ref)*						
Lived with lone parent		−0.207	−0.193		−0.210	−0.195
		(−0.515–0.101)	(−0.493–0.107)		(−0.519–0.099)	(−0.495–0.106)
Lived alone or other living arrangements		−0.059	0.020		−0.040	0.038
		(−0.388–0.271)	(−0.302–0.342)		(−0.371–0.291)	(−0.284–0.361)
*Father upper secondary education (Ref)*						
Father primary education		−0.095	−0.107		−0.098	−0.110
		(−0.297–0.107)	(−0.303–0.089)		(−0.300–0.104)	(−0.307–0.086)
Father tertiary education		0.003	0.023		0.001	0.021
		(−0.242–0.247)	(−0.216–0.261)		(−0.244–0.246)	(−0.218–0.260)
*Current marital status: Married or registered partnership (Ref)*						
Unpartnered			−0.773***			−0.798***
			(−1.211 to −0.336)			(−1.238 to −0.357)
Sep, div, widow			−0.670***			−0.668***
			(−0.879 to −0.462)			(−0.877 to −0.460)
*Suffers from current illness: No (Ref)*						
Suffers from current illness: Yes			−0.261**			−0.252**
			(−0.469 to −0.054)			(−0.460 to −0.044)
*Current employment status: Inactive (Ref)*						
Full-time work			0.154			0.180
			(−0.169–0.476)			(−0.145–0.504)
Part-time paid work			−0.150			−0.142
			(−0.390–0.089)			(−0.382–0.098)
Unemployed			−1.587***			−1.567***
			(−2.706 to −0.468)			(−2.686 to −0.447)
Other situation			−0.544			−0.548
			(−2.115–1.026)			(−2.118–1.022)
Constant	7.402***	7.496***	7.811***	7.566***	7.679***	7.967***
	(6.402–8.402)	(6.492–8.500)	(6.611–9.011)	(6.527–8.604)	(6.635–8.723)	(6.741–9.193)
Observations	1005	1005	1005	1005	1005	1005
*R*-squared	0.042	0.049	0.109	0.047	0.053	0.113

Confidence intervals in parenthesis.**p* < 0.1, ***p* < 0.05, ****p* < 0.01

**Table 9:** Predicted satisfaction with personal relationships. Men. *Source*: Elaboration of the authors based on SHP Biographical files 2002, 2013 and SHP panel (2003–2017)

	Model	Model	Model	Model	Model	Model
	(1)	(2)	(3)	(4)	(5)	(6)
	Gross	Net	Direct	Gross	Net	Direct
*Family trajectory: Traditional (Ref)*						
Late traditional	0.002	-0.002	−0.031	0.047	0.041	0.014
	(−0.191–0.196)	(−0.196–0.192)	(−0.223–0.162)	(−0.174–0.268)	(−0.181–0.263)	(−0.206–0.234)
Childless	−0.288**	−0.285**	−0.139	−0.296**	−0.294**	−0.167
	(−0.507 to −0.069)	(−0.505 to −0.066)	(−0.444–0.166)	(−0.548 to −0.044)	(−0.546 to −0.042)	(−0.488–0.154)
*Work trajectory: Early full-time work (ref)*						
Full-time work after higher education	−0.269*	−0.267*	−0.254*	−0.086	−0.075	−0.072
	(−0.550–0.011)	(−0.550–0.016)	(−0.535–0.027)	(−0.523–0.350)	(−0.513–0.364)	(−0.507–0.363)
Part-time work	−0.104	−0.094	−0.060	−0.150	−0.154	−0.147
	(−0.365–0.156)	(−0.355–0.167)	(−0.320–0.201)	(−0.561–0.260)	(−0.565–0.257)	(−0.554–0.260)
Late traditional* full-time work after higher education				−0.312	−0.322	−0.335
				(−0.904–0.279)	(−0.915–0.270)	(−0.923–0.253)
Late traditional*part-time work				−0.060	−0.033	−0.021
				(−0.685–0.564)	(−0.661–0.595)	(−0.646–0.604)
Childless*full-time work after higher education				−0.286	−0.308	−0.209
				(−1.068–0.497)	(−1.091–0.475)	(−0.991–0.573)
Childless* part-time work				0.201	0.221	0.307
				(−0.422–0.823)	(−0.403–0.845)	(−0.314–0.929)
Age	0.019**	0.019**	0.018*	0.019**	0.019**	0.019*
	(0.004–0.034)	(0.004–0.034)	(−0.000–0.037)	(0.004–0.034)	(0.004–0.034)	(−0.000–0.037)
*Year: 2014 (Ref)*						
Year: 2003	−0.183**	−0.139	−0.141	−0.183**	−0.137	−0.139
	(−0.363 to −0.004)	(−0.330–0.052)	(−0.357–0.075)	(−0.362 to −0.003)	(−0.328–0.055)	(−0.355–0.078)
*Born in Switzerland or Swiss nationality (Ref)*						
Born abroad		−0.164	−0.182*		−0.168	−0.188*
		(−0.365–0.037)	(−0.391–0.026)		(−0.369–0.033)	(−0.397–0.021)
*Lived with both parents (Ref)*						
Lived with lone parent		−0.165	−0.185		−0.168	−0.188
		(−0.440–0.110)	(−0.458–0.089)		(−0.444–0.108)	(−0.463–0.086)
Lived alone or other living arrangements		−0.020	−0.036		−0.026	−0.042
		(−0.383–0.343)	(−0.396–0.324)		(−0.390–0.338)	(−0.404–0.319)
*Father upper secondary education (Ref)*						
Father primary education		−0.005	−0.005		−0.005	−0.005
		(−0.200–0.190)	(−0.198–0.189)		(−0.201–0.190)	(−0.199–0.189)
Father tertiary education		0.046	0.024		0.042	0.018
		(−0.169–0.262)	(−0.190–0.237)		(−0.173–0.258)	(−0.196–0.232)
*Current marital status: Married or registered partnership (Ref)*						
Unpartnered			−0.980***			−0.986***
			(−1.406 to −0.553)			(−1.413 to −0.559)
Sep, div, widow			−0.171			−0.178
			(−0.414–0.072)			(−0.422–0.066)
*Currently have children: Yes (Ref)*						
Currently have children: No			0.193			0.190
			(−0.178–0.564)			(−0.185–0.564)
*Suffers from current illness: No (Ref)*						
Suffers from current illness: Yes			−0.200*			−0.204*
			(−0.405–0.005)			(−0.409–0.002)
*Current employment status: Inactive (Ref)*						
Full-time work			0.060			0.060
			(−0.177–0.298)			(−0.179–0.299)
Part-time paid work			−0.179			−0.186
			(−0.494–0.136)			(−0.502–0.130)
Unemployed			0.008			0.049
			(−0.772–0.789)			(−0.736–0.834)
Constant	7.613***	7.612***	7.747***	7.599***	7.600***	7.736***
	(6.677–8.550)	(6.668–8.556)	(6.480–9.015)	(6.659–8.539)	(6.653–8.547)	(6.465–9.007)
Observations	880	880	880	880	880	880
*R*-squared	0.026	0.031	0.062	0.028	0.033	0.064

Confidence intervals in parenthesis. **p* < 0.1, ***p* < 0.05, ****p* < 0.01

**Table 10:** Predicted satisfaction with personal relationships. Women. *Source*: Elaboration of the authors based on SHP Biographical files 2002, 2013 and SHP panel (2003–2017)

	Model	Model	Model	Model	Model	Model
	(1)	(2)	(3)	(4)	(5)	(6)
	Gross	Net	Direct	Gross	Net	Direct
*Family trajectory: Traditional (Ref)*						
Early Traditional	0.112	0.125	0.125	0.332	0.309	0.285
	(−0.064–0.287)	(−0.052–0.301)	(−0.052–0.303)	(−0.104–0.768)	(−0.130–0.748)	(−0.160–0.729)
Childless	−0.098	−0.121	−0.029	0.017	−0.029	0.051
	(−0.415–0.220)	(−0.439–0.197)	(−0.415–0.357)	(−0.434–0.468)	(−0.482–0.424)	(−0.451–0.553)
*Work trajectory: Full-time work (Ref)*						
Return to part-time	0.036	−0.002	−0.019	0.153	0.096	0.065
	(−0.191–0.263)	(−0.231–0.228)	(−0.257–0.218)	(−0.217–0.524)	(−0.277–0.470)	(−0.314–0.445)
Not in employment	0.132	0.094	0.106	0.313	0.244	0.240
	(−0.132–0.395)	(−0.172–0.359)	(−0.173–0.384)	(−0.086–0.712)	(−0.160–0.648)	(−0.173–0.653)
Early traditional*return to part-time				−0.224	−0.190	−0.159
				(−0.719–0.271)	(−0.686–0.306)	(−0.660–0.341)
Early traditional*not in employment				−0.339	−0.278	−0.245
				(−0.891–0.213)	(−0.833–0.277)	(−0.804–0.314)
Childless*return to part-time				−0.026	−0.002	−0.012
				(−0.739–0.687)	(−0.717–0.713)	(−0.728–0.704)
Childless*not in employment				−0.630	−0.581	−0.592
				(−2.029–0.770)	(−1.981–0.819)	(−1.993–0.809)
Age	0.019**	0.019**	0.027***	0.019**	0.020**	0.027***
	(0.004–0.034)	(0.004–0.034)	(0.010–0.045)	(0.004–0.034)	(0.004–0.035)	(0.009–0.045)
*Year: 2014 (Ref)*						
Year: 2003	−0.130	−0.081	−0.013	−0.136	−0.087	−0.020
	(−0.301–0.041)	(−0.262–0.099)	(−0.210–0.184)	(−0.307–0.036)	(−0.269–0.095)	(−0.218–0.179)
*Born in Switzerland or Swiss nationality (Ref)*						
Born abroad		−0.180*	−0.220**		−0.174*	−0.213**
		(−0.368–0.008)	(−0.411 to −0.029)		(−0.363–0.015)	(−0.406 to −0.020)
Lived with both parents (Ref)						
Lived with lone parent		−0.289**	−0.280*		−0.284*	−0.276*
		(−0.578 to −0.000)	(−0.569–0.009)		(−0.573–0.005)	(−0.565–0.014)
Lived alone or other living arrangements		0.072	0.092		0.059	0.081
		(−0.236–0.381)	(−0.218–0.402)		(−0.251–0.369)	(−0.230–0.393)
*Father upper secondary education (Ref)*						
Father primary education		−0.053	−0.052		−0.053	−0.051
		(−0.242–0.136)	(−0.241–0.137)		(−0.242–0.137)	(−0.241–0.138)
Father tertiary education		−0.015	−0.012		−0.015	−0.010
		(−0.244–0.214)	(−0.242–0.218)		(−0.245–0.215)	(−0.241–0.220)
*Current marital status: Married or registered partnership (Ref)*						
Unpartnered			−0.190			−0.174
			(−0.611–0.231)			(−0.599–0.251)
Sep, div, widow			−0.145			−0.149
			(−0.346–0.056)			(−0.350–0.053)
*Suffers from current illness: No (Ref)*						
Suffers from current illness: Yes			−0.010			−0.010
			(−0.210–0.190)			(−0.210–0.191)
*Current employment status: Inactive (Ref)*						
Full-time work			0.208			0.195
			(−0.103–0.519)			(−0.119–0.508)
Part-time paid work			0.158			0.153
			(−0.073–0.388)			(−0.078–0.385)
Unemployed			−0.118			−0.111
			(−1.196–0.960)			(−1.191–0.969)
Other situation			1.186			1.186
			(−0.327–2.698)			(−0.329–2.701)
Constant	7.474***	7.556***	7.047***	7.345***	7.450***	6.975***
	(6.537–8.410)	(6.616–8.496)	(5.890–8.203)	(6.372–8.319)	(6.472–8.429)	(5.792–8.158)
Observations	1005	1005	1005	1005	1005	1005
*R*-squared	0.014	0.023	0.030	0.016	0.024	0.031

Confidence intervals in parenthesis.**p* < 0.1, ***p* < 0.05, ****p* < 0.01

**Table 11:** Predicted satisfaction with financial situation. Men. *Source*: Elaboration of the authors based on SHP Biographical files 2002, 2013 and SHP panel (2003–2017)

	Model	Model	Model	Model	Model	Model
	(1)	(2)	(3)	(4)	(5)	(6)
	Gross	Net	Direct	Gross	Net	Direct
*Family trajectory: traditional (Ref)*						
Late traditional	0.084	0.067	−0.078	0.070	0.048	−0.088
	(−0.175–0.343)	(−0.186–0.320)	(−0.333–0.176)	(−0.226–0.366)	(−0.241–0.337)	(−0.378–0.202)
Childless	−0.068	−0.053	−0.507**	−0.091	−0.091	−0.513**
	(−0.361–0.225)	(−0.339–0.233)	(−0.914 to −0.099)	(−0.428–0.245)	(−0.419–0.238)	(−0.941 to −0.085)
*Work trajectory: early full-time work (ref)*						
Full−time work after higher education	0.330*	0.394**	0.188	0.485	0.570*	0.290
	(−0.046–0.705)	(0.024–0.763)	(−0.186–0.563)	(−0.099–1.069)	(−0.001–1.141)	(−0.292–0.871)
Part-time work	−0.124	−0.091	0.025	−0.356	−0.383	−0.067
	(−0.472–0.224)	(−0.431–0.249)	(−0.319–0.370)	(−0.905–0.193)	(−0.918–0.153)	(−0.606–0.472)
Late traditional* full-time work after higher education				−0.174	−0.194	−0.003
				(−0.965–0.618)	(−0.966–0.578)	(−0.782–0.777)
Late traditional*part-time work				0.303	0.362	0.024
				(−0.532–1.138)	(−0.457–1.181)	(−0.802–0.849)
Childless*full-time work after higher education				−0.461	−0.528	−0.596
				(−1.507–0.586)	(−1.549–0.492)	(−1.633–0.440)
Childless* part-time work				0.448	0.586	0.250
				(−0.384–1.281)	(−0.227–1.400)	(−0.578–1.078)
Age	0.042***	0.044***	0.041***	0.042***	0.044***	0.041***
	(0.022–0.062)	(0.024–0.063)	(0.015–0.066)	(0.022–0.062)	(0.024–0.063)	(0.015–0.066)
*Year: 2014 (Ref)*						
Year: 2003	0.165	0.387***	0.285*	0.162	0.385***	0.291**
	(−0.075–0.405)	(0.137–0.636)	(−0.003–0.573)	(−0.078–0.402)	(0.136–0.634)	(0.002–0.579)
*Born in Switzerland or Swiss nationality (Ref)*						
Born abroad		−0.771***	−0.685***		−0.777***	−0.689***
		(−1.033 to −0.509)	(−0.963 to −0.407)		(−1.039 to −0.515)	(−0.968 to −0.410)
*Lived with both parents (Ref)*						
Lived with lone parent		−0.414**	−0.388**		−0.431**	−0.389**
		(−0.773 to −0.056)	(−0.750 to −0.027)		(−0.792 to −0.071)	(−0.753 to −0.025)
Lived alone or other living arrangements		−0.209	−0.237		−0.207	−0.237
		(−0.683–0.264)	(−0.720–0.245)		(−0.681–0.268)	(−0.722–0.248)
*Father upper secondary education (Ref)*						
Father primary education		−0.274**	−0.204		−0.278**	−0.211
		(−0.528 to −0.020)	(−0.461–0.052)		(−0.533 to −0.024)	(−0.468–0.046)
Father tertiary education		−0.254*	−0.345**		−0.262*	−0.346**
		(−0.535–0.027)	(−0.627 to −0.062)		(−0.544–0.019)	(−0.629 to −0.062)
*Current marital status: Married or registered partnership (Ref)*						
Unpartnered			0.031			0.044
			(−0.539–0.602)			(−0.527–0.615)
Sep, div, widow			−0.168			−0.171
			(−0.489–0.154)			(−0.493–0.151)
*Currently have children: Yes (Ref)*						
Currently have children: No			0.497*			0.526**
			(−0.007–1.001)			(0.017–1.035)
*Suffers from current illness: No (Ref)*						
Suffers from current illness: Yes			−0.106			−0.107
			(−0.377–0.166)			(−0.380–0.166)
*Current employment status: Inactive (Ref)*						
Full-time work			−0.074			−0.067
			(−0.395–0.247)			(−0.390–0.256)
Part-time paid work			0.094			0.101
			(−0.324–0.513)			(−0.320–0.522)
Unemployed			−1.592***			−1.606***
			(−2.694 to −0.490)			(−2.712 to −0.500)
Net personal income			0.003***			0.003***
			(0.002–0.004)			(0.002–0.004)
Constant	5.262***	5.445***	5.598***	5.272***	5.467***	5.600***
	(4.009–6.515)	(4.214–6.676)	(3.905–7.291)	(4.015–6.529)	(4.232–6.701)	(3.903–7.297)
Observations	880	880	816	880	880	816
*R*-squared	0.025	0.077	0.115	0.027	0.081	0.118

Confidence intervals in parenthesis.**p* < 0.1, ***p* < 0.05, ****p* < 0.01

**Table 12:** Predicted satisfaction with financial situation. Women. *Source*: Elaboration of the authors based on SHP Biographical files 2002, 2013 and SHP panel (2003–2017)

	Model	Model	Model	Model	Model	Model
	(1)	(2)	(3)	(4)	(5)	(6)
	Gross	Net	Direct	Gross	Net	Direct
*Family trajectory: traditional (Ref)*						
Early Traditional	−0.195	−0.146	−0.082	−0.577*	−0.632*	−0.443
	(−0.457–0.068)	(−0.407–0.116)	(−0.352–0.188)	(−1.227–0.074)	(−1.279–0.014)	(−1.107–0.222)
Childless	−0.801***	−0.880***	−0.446	−1.115***	−1.232***	−0.522
	(−1.277 to −0.324)	(−1.352 to −0.408)	(−1.007–0.114)	(−1.790 to −0.440)	(−1.902 to −0.561)	(−1.260–0.215)
*Work trajectory: full−time work (Ref)*						
Return to part−time	−0.083	−0.227	−0.157	−0.476*	−0.667**	−0.428
	(−0.423–0.256)	(−0.567–0.112)	(−0.512–0.198)	(−1.028–0.077)	(−1.218 to −0.117)	(−0.997–0.140)
Not in employment	0.114	−0.010	0.150	−0.014	−0.223	0.091
	(−0.281–0.509)	(−0.403–0.383)	(−0.287–0.587)	(−0.609–0.581)	(−0.819–0.372)	(−0.555–0.738)
Early traditional*return to part-time				0.591	0.689*	0.515
				(−0.147–1.328)	(−0.042–1.420)	(−0.229–1.260)
Early traditional*not in employment				0.202	0.377	0.245
				(−0.621–1.024)	(−0.441–1.194)	(−0.613–1.102)
Childless*return to part-time				0.985*	0.997*	0.427
				(−0.080–2.050)	(−0.059–2.052)	(−0.603–1.457)
Childless*not in employment				−1.871*	−1.930*	−2.500**
				(−3.959–0.216)	(−3.994–0.133)	(−4.483 to −0.518)
Age	0.026**	0.027**	0.033**	0.025**	0.025**	0.031**
	(0.004–0.049)	(0.004–0.049)	(0.006–0.061)	(0.002–0.047)	(0.003–0.047)	(0.004–0.058)
*Year: 2014 (Ref)*						
Year: 2003	0.249*	0.408***	0.329**	0.253*	0.420***	0.332**
	(−0.007–0.506)	(0.140–0.676)	(0.025–0.633)	(−0.003–0.509)	(0.152–0.689)	(0.028–0.637)
*Born in Switzerland or Swiss nationality (Ref)*						
Born abroad		−0.638***	−0.520***		−0.659***	−0.536***
		(−0.917 to −0.359)	(−0.812 to −0.228)		(−0.939 to −0.380)	(−0.829 to −0.243)
*Lived with both parents (Ref)*						
Lived with lone parent		−0.252	−0.285		−0.234	−0.258
		(−0.679–0.175)	(−0.720–0.150)		(−0.660–0.193)	(−0.692–0.176)
Lived alone or other living arrangements		0.135	0.228		0.155	0.243
		(−0.322–0.592)	(−0.227–0.684)		(−0.302–0.612)	(−0.213–0.698)
*Father upper secondary education (Ref)*						
Father primary education		−0.258*	−0.194		−0.255*	−0.195
		(−0.538–0.022)	(−0.482–0.095)		(−0.535–0.024)	(−0.483–0.093)
Father tertiary education		0.209	0.170		0.204	0.176
		(−0.130–0.548)	(−0.176–0.515)		(−0.135–0.543)	(−0.169–0.521)
*Current marital status: Married or registered partnership (Ref)*						
Unpartnered			−1.059***			−1.101***
			(−1.670 to −0.449)			(−1.713 to −0.489)
Sep, div, widow			−1.200***			−1.204***
			(−1.523 to −0.878)			(−1.526 to −0.882)
*Suffers from current illness: No (Ref)*						
Suffers from current illness: Yes			−0.111			−0.102
			(−0.411–0.188)			(−0.400–0.196)
*Current employment status: Inactive (Ref)*						
Full-time work			−0.076			−0.078
			(−0.567–0.415)			(−0.570–0.414)
Part-time paid work			−0.204			−0.202
			(−0.562–0.154)			(−0.559–0.155)
Unemployed			−0.935			−0.928
			(−2.599–0.729)			(−2.588–0.732)
Other situation			−0.536			−0.507
			(−3.135–2.063)			(−3.098–2.083)
Net personal income			0.016***			0.016***
			(0.012–0.021)			(0.012–0.021)
Constant	6.378***	6.629***	5.853***	6.699***	7.026***	6.162***
	(4.975–7.781)	(5.236–8.021)	(4.020–7.687)	(5.246–8.152)	(5.582–8.469)	(4.300–8.023)
Observations	1003	1003	868	1003	1003	868
*R*-squared	0.024	0.053	0.134	0.033	0.063	0.144

Confidence intervals in parenthesis.**p* < 0.1, ***p* < 0.05, ****p* < 0.01

**Table 13:** Predicted life satisfaction. Robustness check with pre-trajectories health conditions. Women and men. *Source*: Elaboration of the authors based on SHP Biographical files 2013 and SHP panel (2014–2017)

	Model	Model	Model	Model	Model	Model
	(1)	(2)	(3)	(4)	(5)	(6)
	Net	Direct	Direct, Interaction	Net	Direct	Direct, Interaction
	Men			Women		
*Family trajectory: traditional (Ref)*						
Late traditional	0.194	0.148	0.280*			
	(−0.055–0.444)	(−0.100–0.397)	(−0.012–0.572)			
Childless	0.170	0.186	0.138			
	(−0.125–0.465)	(−0.297–0.669)	(−0.363–0.639)			
*Work trajectory: full−time work (Ref)*						
Full−time work after Higher education	0.159	0.146	0.346			
	(−0.127–0.445)	(−0.138–0.430)	(−0.089–0.782)			
Part-time work	−0.203	−0.162	−0.192			
	(−0.622–0.216)	(−0.586–0.262)	(−0.805–0.422)			
*Family trajectory: traditional (Ref)*						
Early traditional				0.164	0.183	−0.199
				(−0.087–0.415)	(−0.063–0.429)	(−0.757–0.359)
Childless				−0.463**	−0.192	−0.758**
				(−0.897 to −0.029)	(−0.700–0.315)	(−1.406 to −0.109)
*Work trajectory: full−time work (Ref)*						
Return to part-time				−0.204	−0.266*	−0.683***
				(−0.514–0.106)	(−0.579–0.047)	(−1.159 to −0.208)
Not in employment				−0.159	−0.325	−0.653**
				(−0.552–0.234)	(−0.728–0.079)	(−1.244 to −0.063)
Late traditional* full−time work after higher education			−0.448			
			(−1.056–0.160)			
Late traditional*part−time work			−0.549			
			(−1.579–0.482)			
Childless*full−time work after higher education			−0.071			
			(−0.874–0.732)			
Childless* part−time work			0.589			
			(−0.395–1.573)			
Early traditional*return to part−time						0.516
						(−0.117–1.149)
Early traditional*not in employment						0.365
						(−0.416–1.146)
Childless*return to part−time						1.173**
						(0.266–2.080)
Childless*not in employment						2.432*
						(−0.292–5.157)
Age	0.033***	0.039***	0.035**	0.028***	0.026**	0.026**
	(0.013–0.053)	(0.011–0.067)	(0.007–0.064)	(0.007–0.049)	(0.001–0.052)	(0.000–0.051)
*Born in Switzerland or Swiss nationality (Ref)*						
Born abroad	−0.139	−0.123	−0.124	−0.118	−0.145	−0.167
	(−0.466–0.188)	(−0.449–0.203)	(−0.449–0.202)	(−0.425–0.188)	(−0.444–0.154)	(−0.466–0.132)
*Lived with both parents (Ref)*						
Lived with lone parent	−0.223	−0.083	−0.049	−0.196	−0.133	−0.155
	(−0.587–0.142)	(−0.452–0.286)	(−0.419–0.321)	(−0.630–0.238)	(−0.554–0.287)	(−0.574–0.265)
Lived alone or other living arrangements	−0.062	−0.003	0.014	0.324	0.425*	0.435*
	(−0.545–0.421)	(−0.480–0.475)	(−0.463–0.491)	(−0.146–0.794)	(−0.033–0.882)	(−0.021–0.892)
*Father upper secondary education (Ref)*						
Father primary education	−0.193	−0.164	−0.167	−0.136	−0.141	−0.134
	(−0.441–0.054)	(−0.409–0.080)	(−0.413–0.078)	(−0.401–0.128)	(−0.397–0.115)	(−0.390–0.123)
Father tertiary education	0.051	0.077	0.061	−0.009	0.020	0.002
	(−0.248–0.349)	(−0.219–0.373)	(−0.235–0.357)	(−0.331–0.313)	(−0.294–0.334)	(−0.311–0.316)
*Health issues before age 20: No (Ref)*						
Health issues before age 20: Yes	−0.080	−0.078	−0.078	0.015	0.006	−0.015
	(−0.312–0.152)	(−0.309–0.153)	(−0.310–0.154)	(−0.238–0.267)	(−0.239–0.252)	(−0.260–0.229)
*Suffers from current illness: No (Ref)*						
Suffers from current illness: Yes		−0.091	−0.091		−0.178	−0.195
		(−0.371–0.189)	(−0.371–0.189)		(−0.457–0.100)	(−0.473–0.084)
*Current marital status: Married or registered partnership (Ref)*						
Unpartnered		−0.311	−0.327		−0.686**	−0.697**
		(−0.829–0.208)	(−0.845–0.190)		(−1.243 to −0.129)	(−1.257 to −0.137)
Sep, div, widow		−0.206	−0.210		−0.731***	−0.724***
		(−0.518–0.106)	(−0.522–0.101)		(−1.016 to −0.446)	(−1.009 to −0.440)
*Currently have children: Yes (Ref)*						
Currently have children: No		0.171	0.164			
		(−0.363–0.706)	(−0.374–0.701)			
*Current employment status: Inactive (Ref)*						
Full−time work		0.195	0.166		0.259	0.248
		(−0.145–0.534)	(−0.177–0.509)		(−0.164–0.681)	(−0.173–0.670)
Part−time paid work		−0.171	−0.202		−0.079	−0.096
		(−0.591–0.249)	(−0.625–0.221)		(−0.382–0.225)	(−0.398–0.207)
Unemployed		−1.608***	−1.586***		−2.338***	−2.422***
		(−2.581 to −0.635)	(−2.559 to −0.612)		(−3.876 to −0.801)	(−3.956 to −0.888)
Other situation					0.415	0.492
					(−2.215–3.044)	(−2.128–3.112)
Constant	6.547***	6.174***	6.374***	6.993***	7.402***	7.787***
	(5.286–7.808)	(4.290–8.059)	(4.474–8.274)	(5.686–8.300)	(5.726–9.078)	(6.086–9.489)
Observations	474	474	474	540	540	540
*R*-squared	0.050	0.095	0.106	0.037	0.113	0.128

Confidence intervals in parenthesis. **p* < 0.1, ***p* < 0.05, ****p* < 0.01

## Footnotes

1 The vast majority of respondents participated in the first wave after the biographical calendar was collected. Respectively, 14 (0.6%) and 54 (2.3%) respondents did not participate in the 2003 and 2014 waves but in the subsequent ones.

2 Missing data on control variables (*N* = 414) are mostly due to missing information on parental education (*N* = 362) and marital status (*N* = 39).

3 The question formulations are as follows. Life satisfaction: “*In general, how satisfied are you with your life if 0 means ‘not at all satisfied’ and 10 means ‘completely satisfied’?*”; Satisfaction with personal relationships: “*How satisfied are you with your personal, social and family relationships, if 0 means ‘not at all satisfied’ and 10 ‘completely satisfied’?*” and satisfaction with financial situation: “*Overall how satisfied are you with your financial situation, if 0 means ‘not at all satisfied’ and 10 ‘completely satisfied’?*”.

4 Here, age also measures the time between the end of the trajectory and when wellbeing is recorded.

5 No women in our sample had children after the age of 50.

6 Robustness checks have been conducted using simple Hamming distance and longest common subsequence (LCS). Very small differences emerge in the family clusters, because, with respect to both Hamming measures, LCS tends to stress distance between sequences linked to state duration more and timing less. We preferred to highlight timing distances between trajectories as we believe the plan of temporal structure of family formation is crucial in contemporary societies (Aassve et al., 2007b); Oechsle and Geissler, 2003). Partitioning Around Medoids (PAM) as clustering method produces qualitatively identical clusters.

7 We follow previous studies (Ferrer-i-Carbonell and Frijters, 2004) showing that the assumptions of cardinality or ordinality have substantially no impact on empirical results and assume the cardinality of satisfaction measures.

8 The Chi-squared test allows us to reject the hypothesis of independence of the two variables ($\chi ,$ ^2^ = 26.35, *p* = 0.000).

9 Weighted distributions are essentially identical (available upon request).

10 Based on the net model. Results do not differ substantially if we plot predicted life satisfaction from the direct model.

11 We find no difference in the association between family trajectories with early (for women) *versus* late (for men) family formation and wellbeing measures so, for simplicity, the figures report only presents the associations for the traditional transitions. Traditional transitions for both men and women mean family formation takes place round their mid-20 s.
