# Correction to: Joint Family and Work Trajectories and Multidimensional Wellbeing

**DOI:** 10.1007/s10680-022-09615-6

**Published:** 2022-03-23

**Authors:** C. L. Comolli, L. Bernardi, M. Voorpostel

**Affiliations:** 1 University of Lausanne, Lausanne, Switzerland; 2 FORS (Swiss Centre of Expertise in the Social Sciences), Lausanne, Switzerland

## Correction to: European Journal of Population (2021) 37:643–696 https://doi.org/10.1007/s10680-021-09583-3

The Funding information section was missing from this article and should have read:

The project was supported by the Swiss National Science Foundation (Grant 100017_182301, project WELLWAYS). This study has been realized using the data collected by the Swiss Household Panel (SHP), which is based at FORS-the Swiss Centre of Expertise in the Social Sciences.

The original article has been corrected.

